# Supplementary material for: Individual environmental niches in mobile organisms
Source: Nat Commun. 2021 Jul 27;12:4572. doi: 10.1038/s41467-021-24826-x (PMC8316569; doi:10.1038/s41467-021-24826-x)
Supplement: Supplementary file 1 — Supplementary Information [file 41467_2021_24826_MOESM1_ESM.pdf]

Supplementary Information for Individual Environmental Niches in Mobile Organisms

Supplementary Table 1. Environmental variables used as part of resource selection and niche analysis. We chose variables that we hypothesized storks should show preference for and thus should be Grinnellian foraging niche axes.

16-day NDVI	Fusion of Landsat 7 & 8 Collection 1 surface reflectance[1,2]. Landsat 7 & 8 NDVI was harmonized following Roy et al.[3].
Percent tree cover	Percentage of each 30 meter pixel that is forested[4]. Downloaded from https://glad.umd.edu/dataset/global-2010-bare-ground-30-m
Percent bare ground	Percentage of each 30 meter pixel that is bare ground[4]. Downloaded from https://glad.umd.edu/dataset/global-2010-tree-cover-30-m
Distance to urban	Distance from each pixel to the nearest pixel designated as urban. Derived from the 12 m Global Urban Footprint product[5].
Distance to forest	Distance from each pixel to the nearest pixel that has > 30 % forest. Derived from the percent tree cover product[4].

Beuster 2013

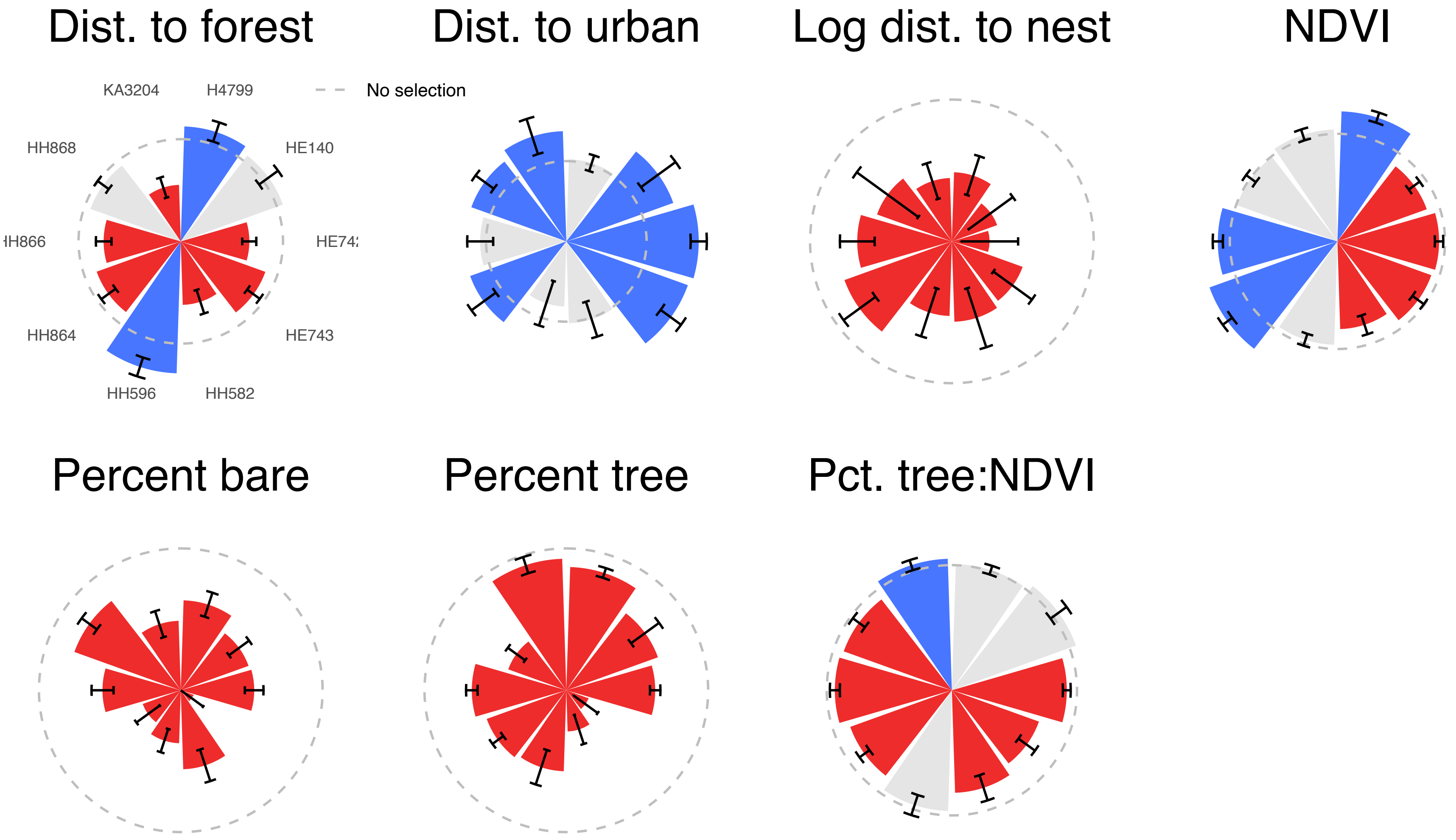

Beuster 2014

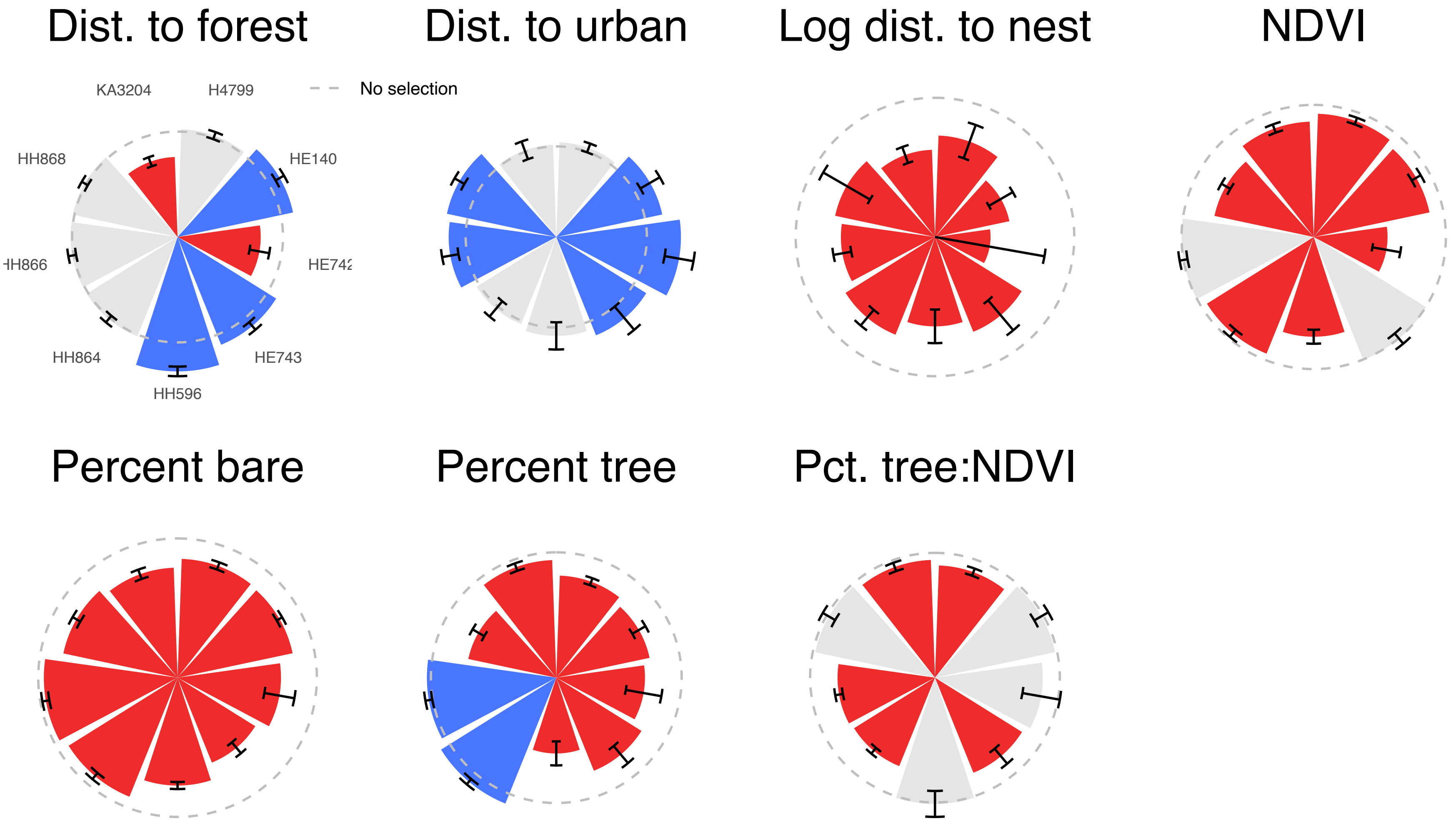

Beuster 2015

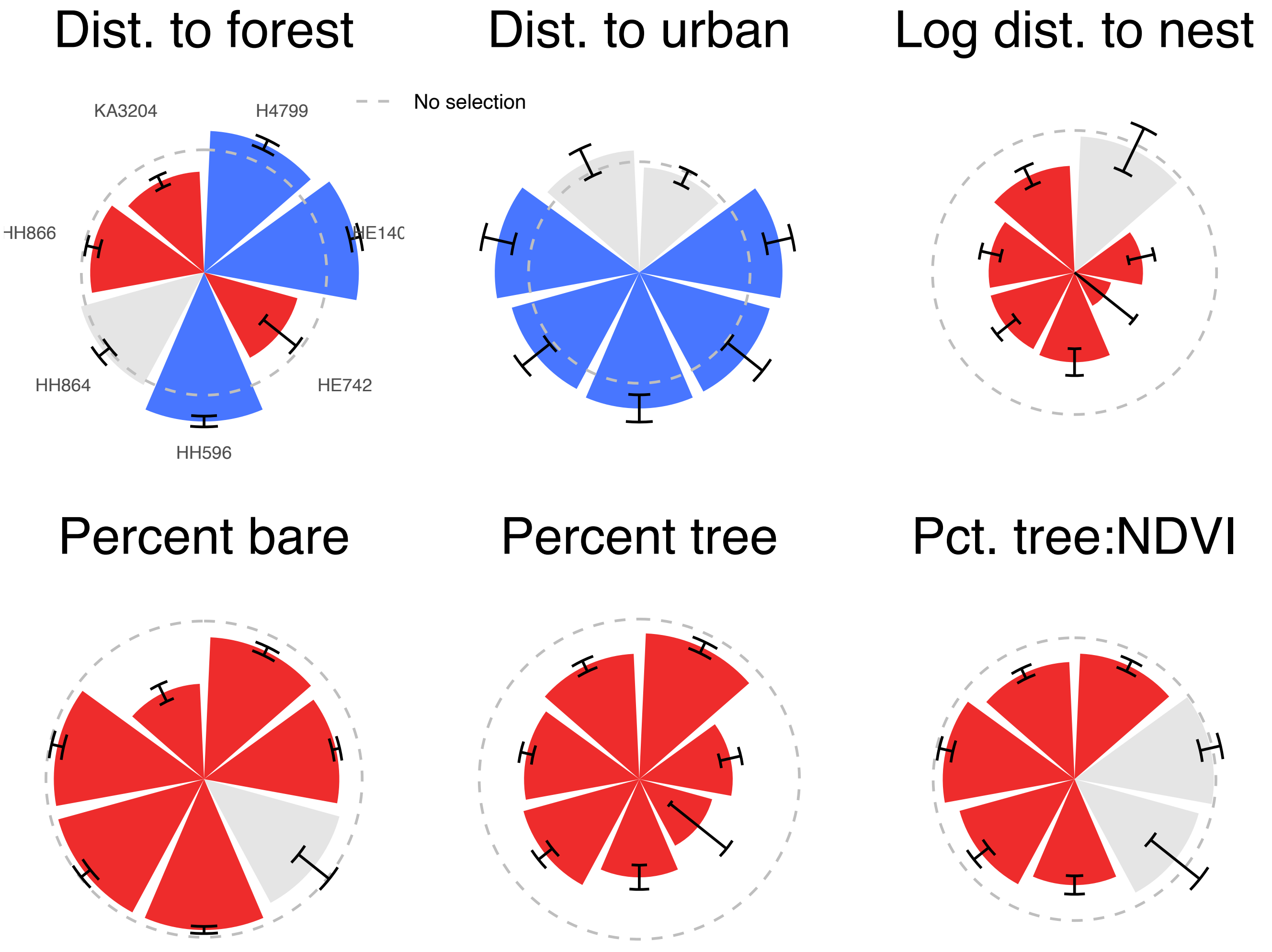

Beuster 2016

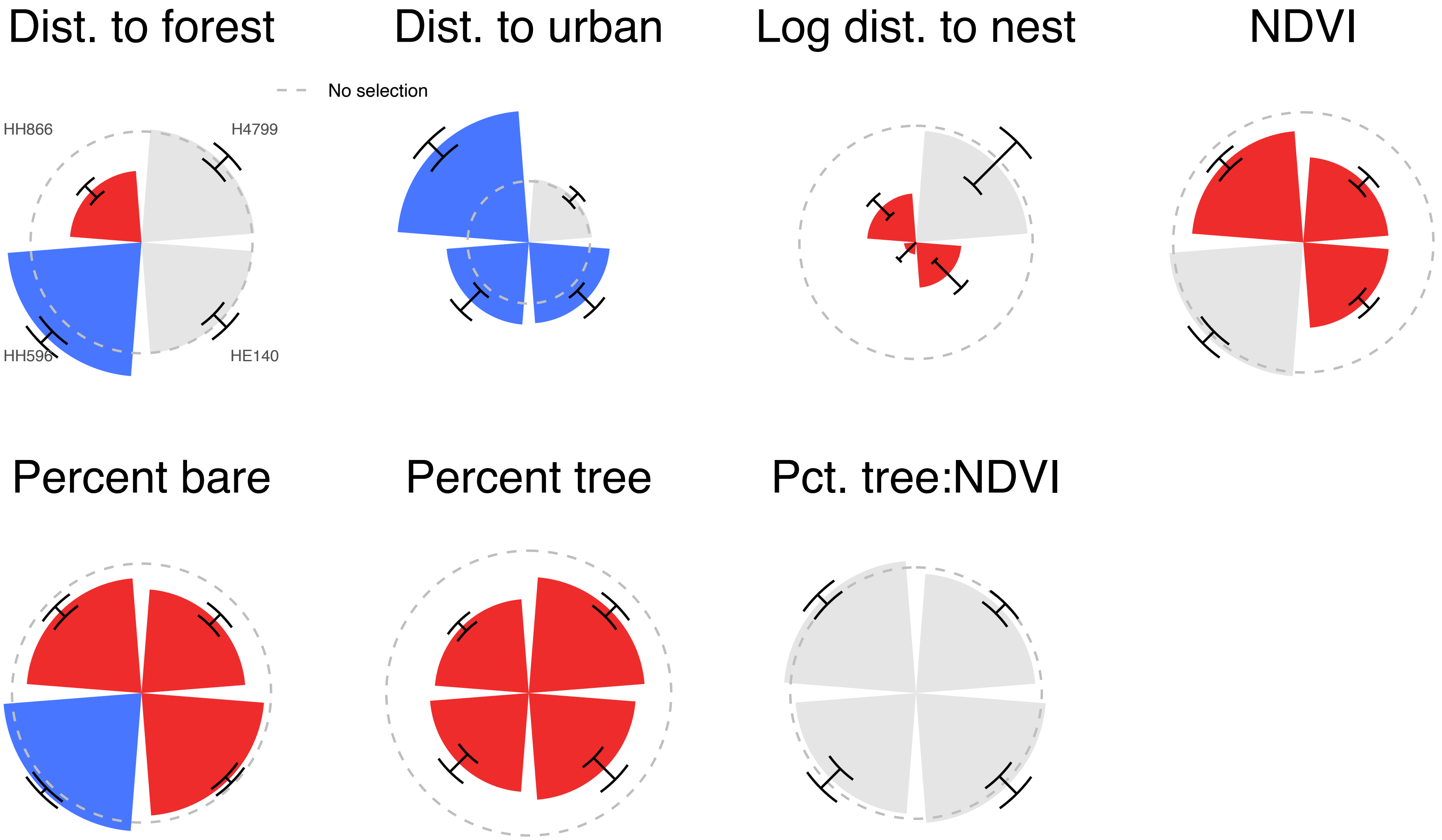

Dromling 2013

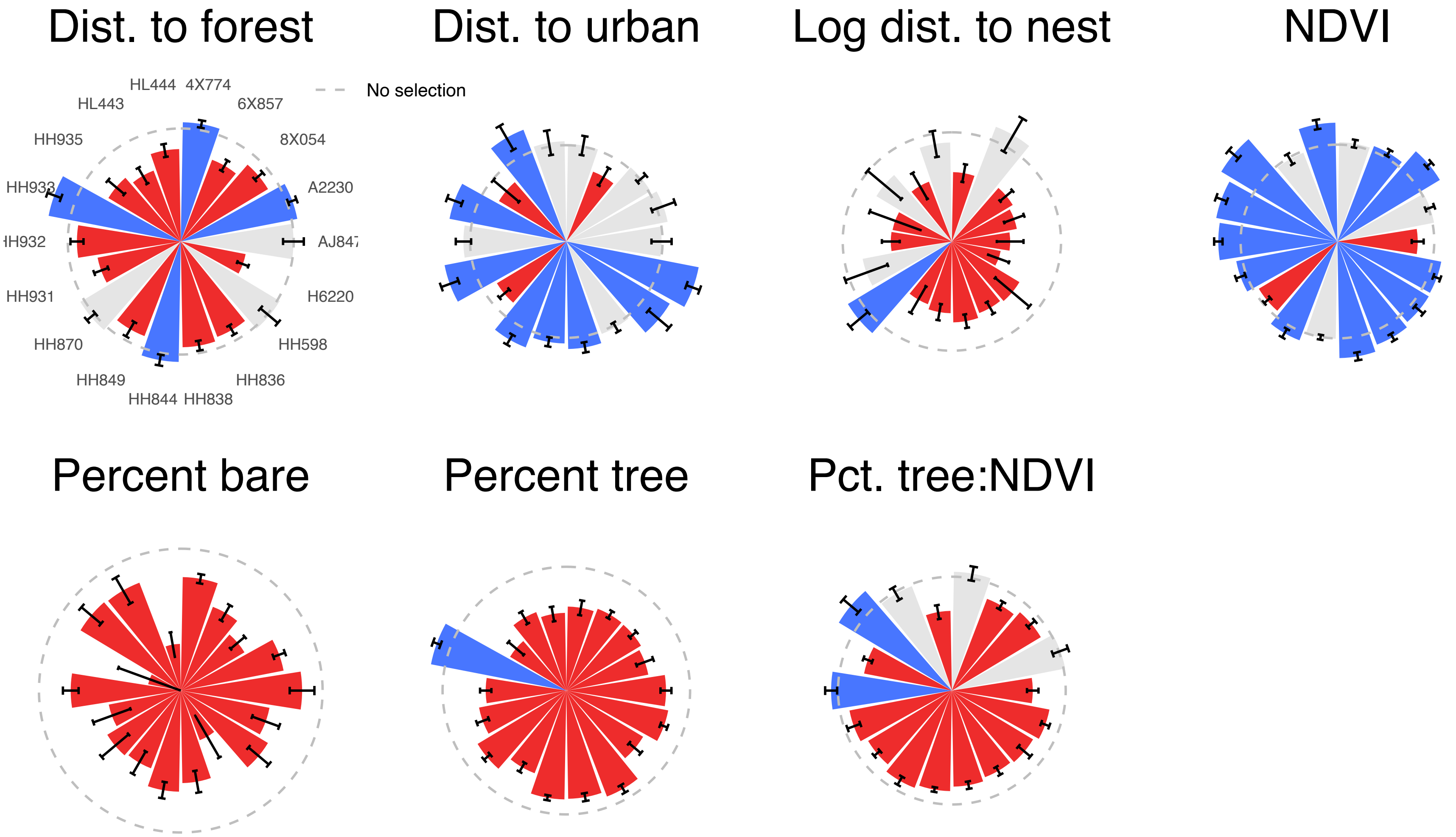

Dromling 2014

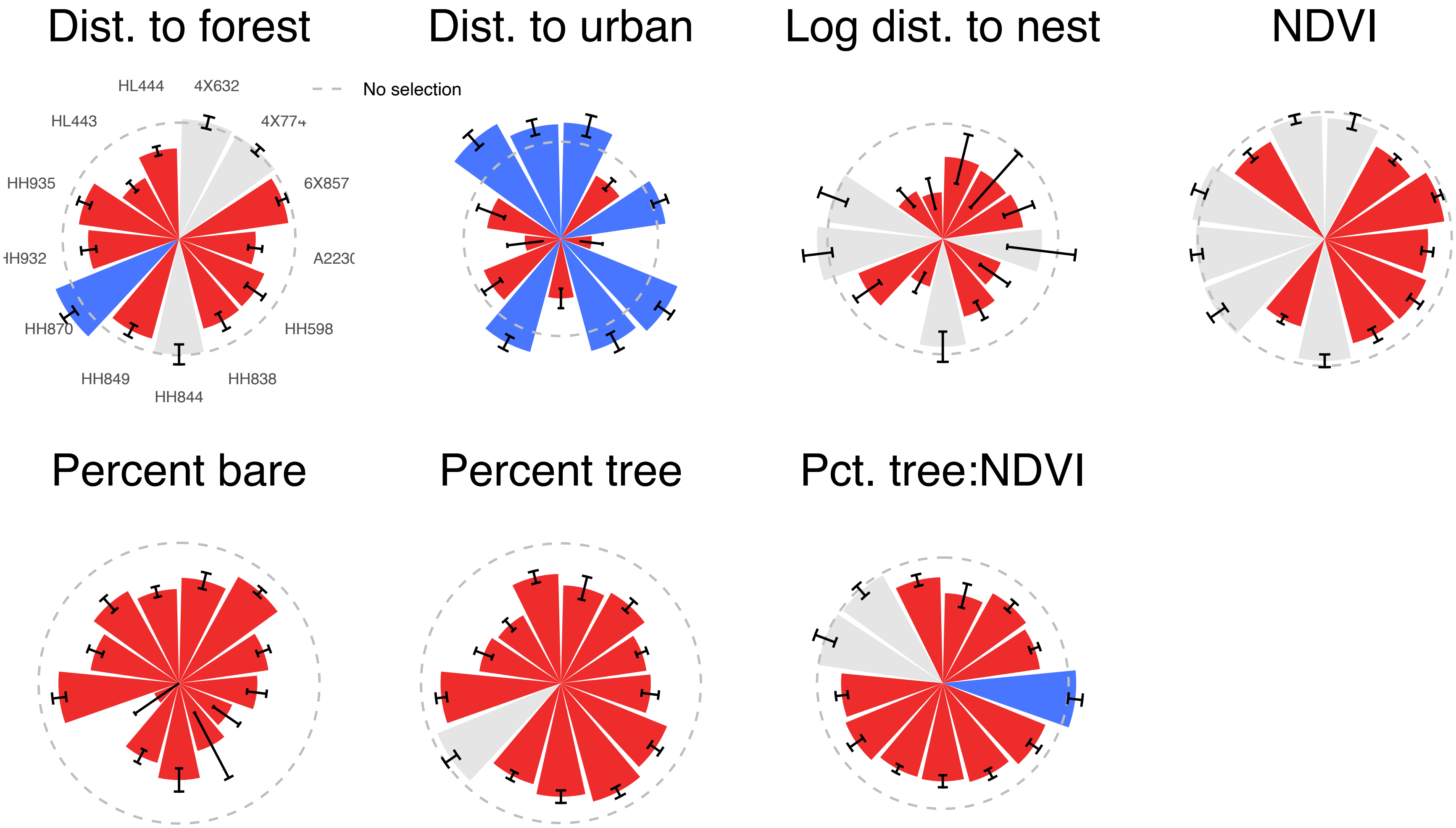

Dromling 2015

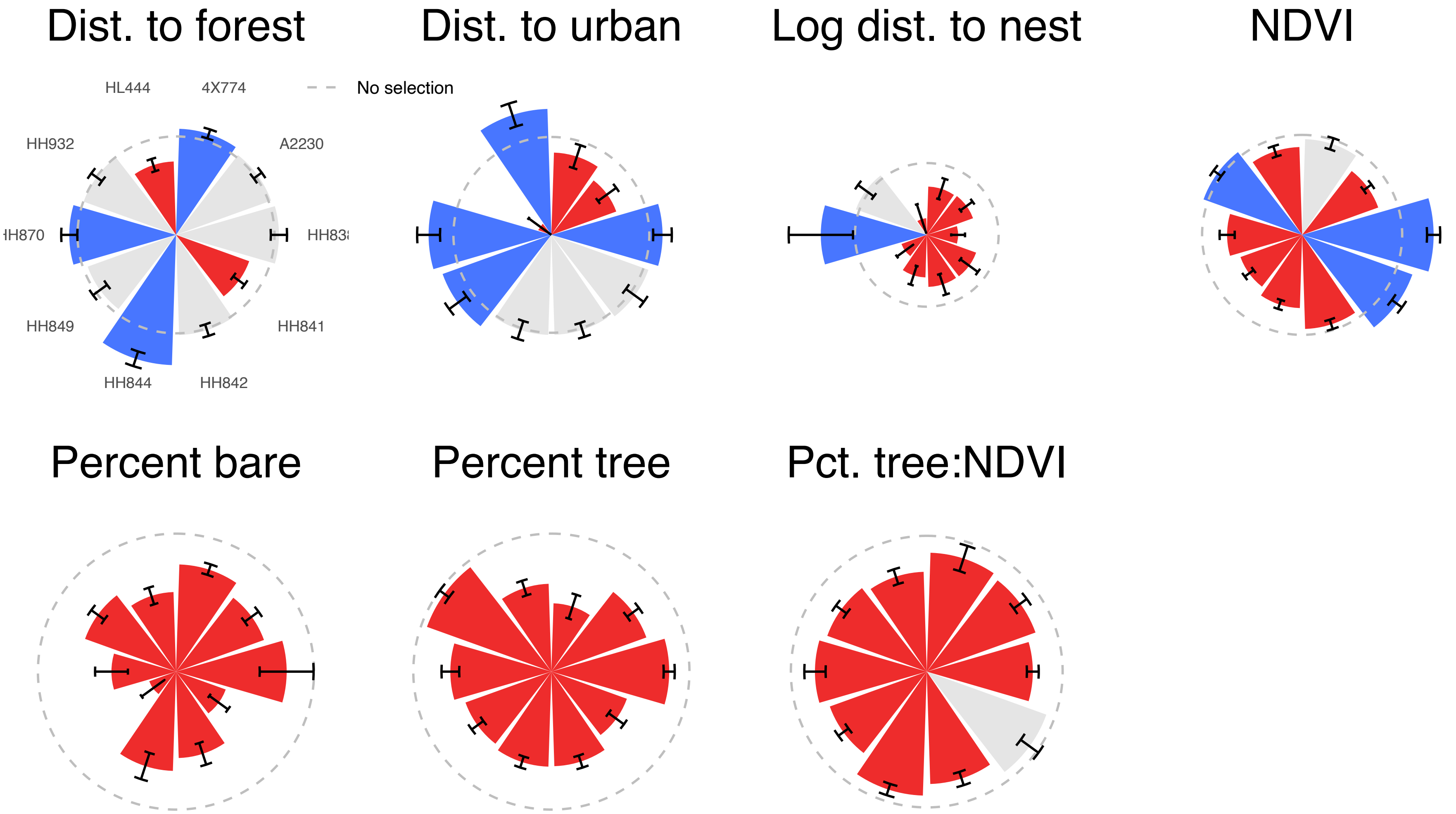

Dromling 2016

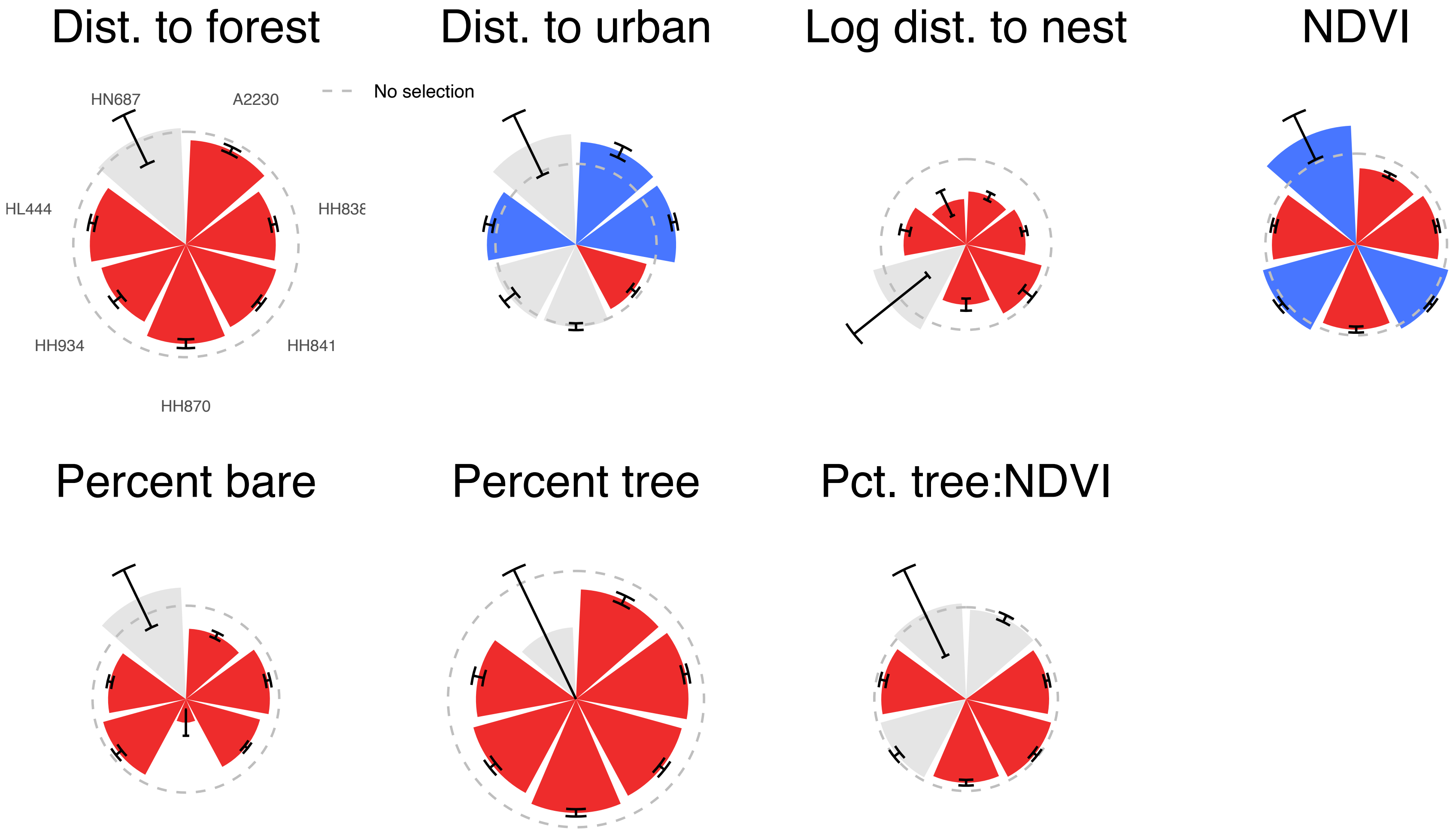

Loburg 2013

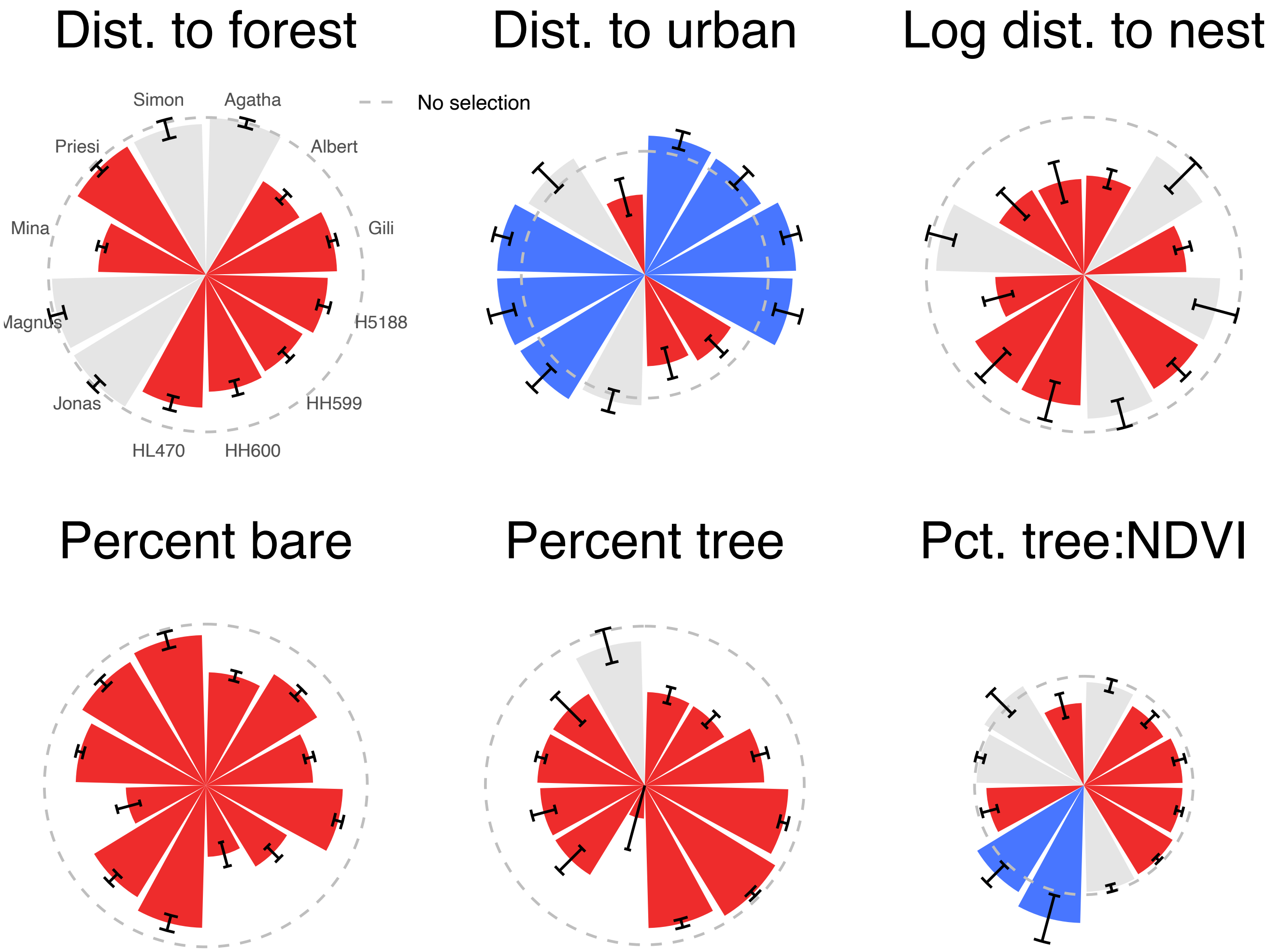
Dist. to forest
Dist. to urban
Log dist. to nest
NDVI
No selection
Simon
Agatha
Albert
Gili
H5188
HH599
HH600
HL470
Jonas
Magnus
Mina
Priesi
Percent bare
Percent tree
Pct. tree:NDVI

Loburg 2014

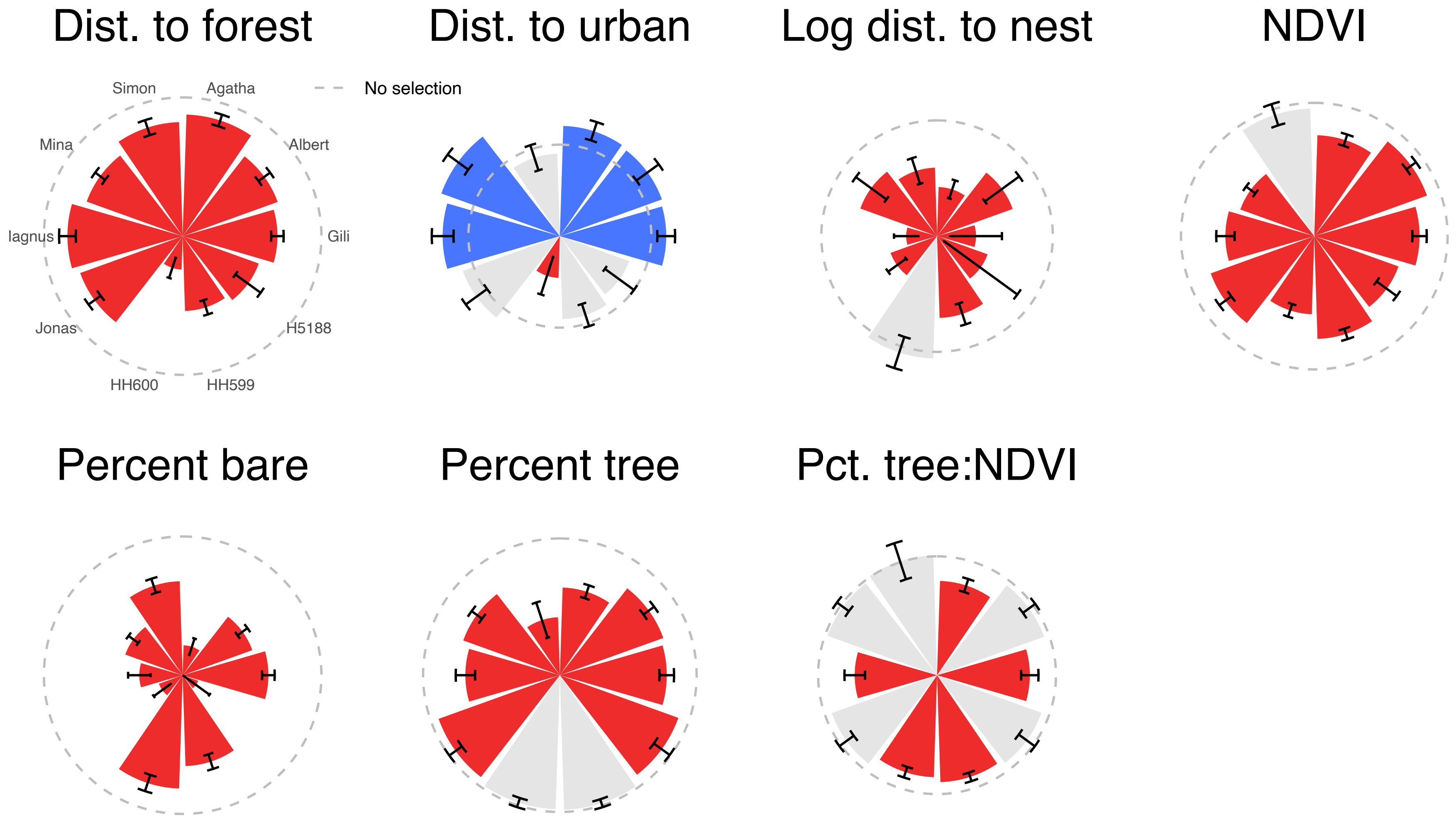
Dist. to forest
Dist. to urban
Log dist. to nest
NDVI
Simon
Agatha
No selection
Mina
Albert
Iagnus
Gili
Jonas
H5188
HH600
HH599
Percent bare
Percent tree
Pct. tree:NDVI

Loburg 2015

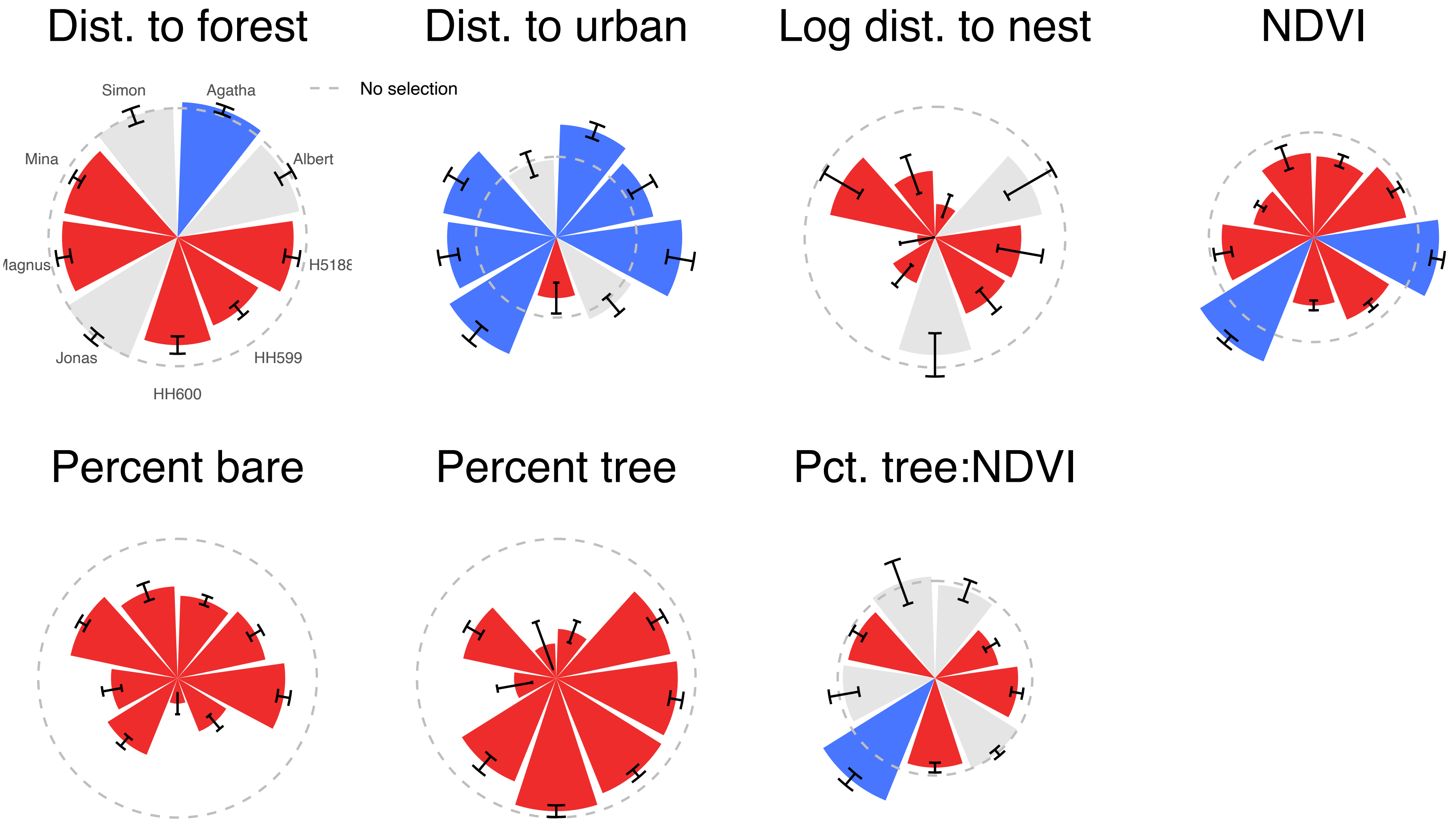
Dist. to forest
Dist. to urban
Log dist. to nest
NDVI
Simon
Agatha
No selection
Mina
Albert
lagnus
H518
Jonas
HH599
HH600
Percent bare
Percent tree
Pct. tree:NDVI

Loburg 2016

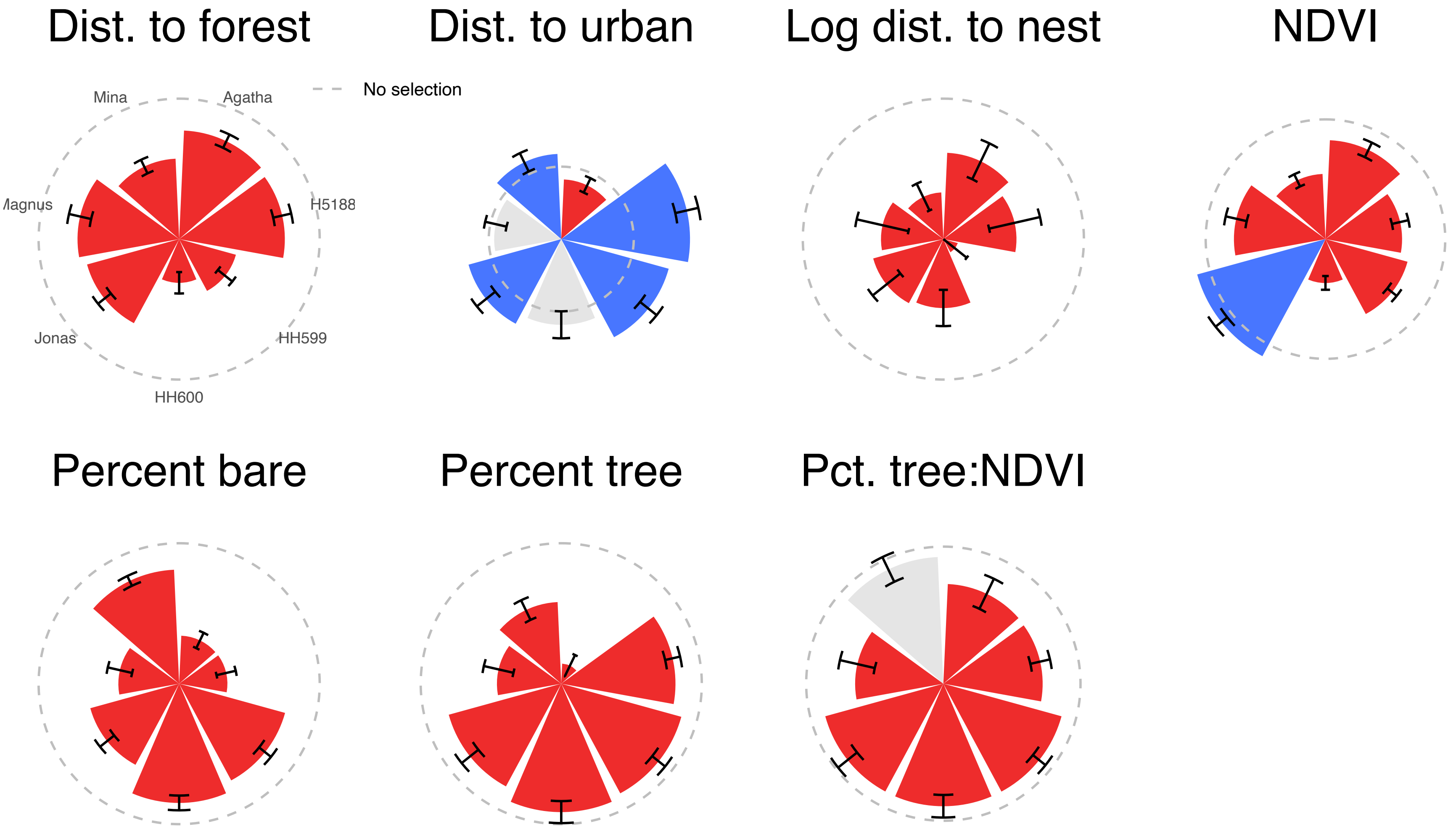
Dist. to forest
Dist. to urban
Log dist. to nest
NDVI
Mina
Agatha
No selection
H5188
Jonas
HH599
HH600
Percent bare
Percent tree
Pct. tree:NDVI

Supplementary Figure 1. Parameter values and confidence intervals from step selection analysis for three populations (Beuster, Drömling, Loburg) for breeding seasons 2013–2016. Covariates include the five environmental variables (Table S1) as well as a covariate for distance to the nest and an interaction term for percent forest and NDVI. Individual bars show mean selection coefficients for a given variable and individual combination, scaled from zero to one. Black error bars represent upper and lower 95% confidence intervals for the mean selection coefficient value. Coefficients significantly larger than zero (blue bars) indicate an individual positively associated with the variable relative to availability. Coefficients significantly smaller than zero (red bars) indicate avoidance of conditions represented by the variable, relative to availability. Grey bars indicate no significant associations.

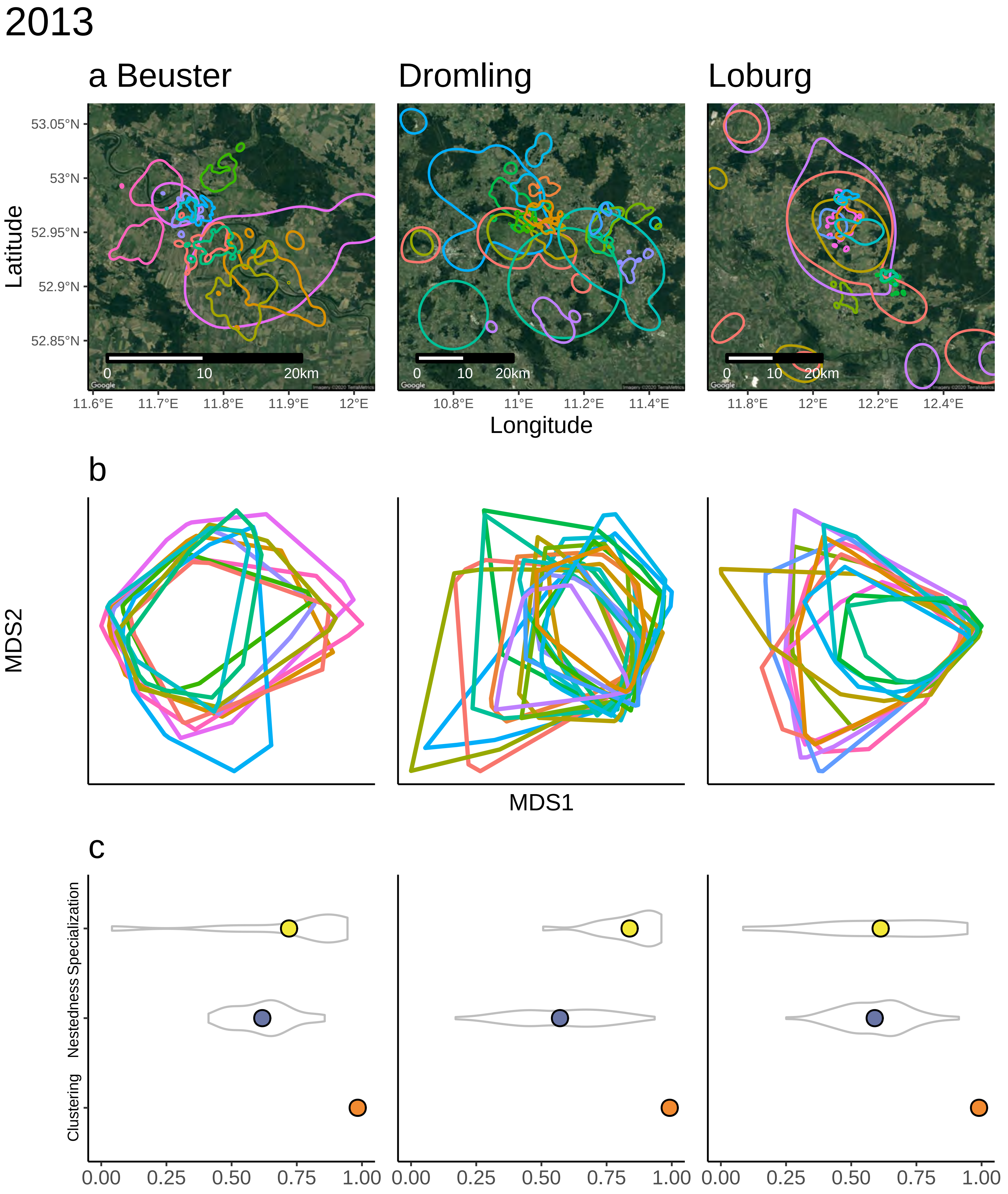
2013
a Beuster
Dromling
Loburg
Latitude
53.05°N
53°N
52.95°N
52.9°N
52.85°N
0
10
20km
Google
11.6°E
11.7°E
11.8°E
11.9°E
12°E
10.8°E
11°E
11.2°E
11.4°E
11.8°E
12°E
12.2°E
12.4°E
Longitude
b
MDS2
MDS1
c
Specialization
Nestedness
Clustering
0.00
0.25
0.50
0.75
1.00

2014

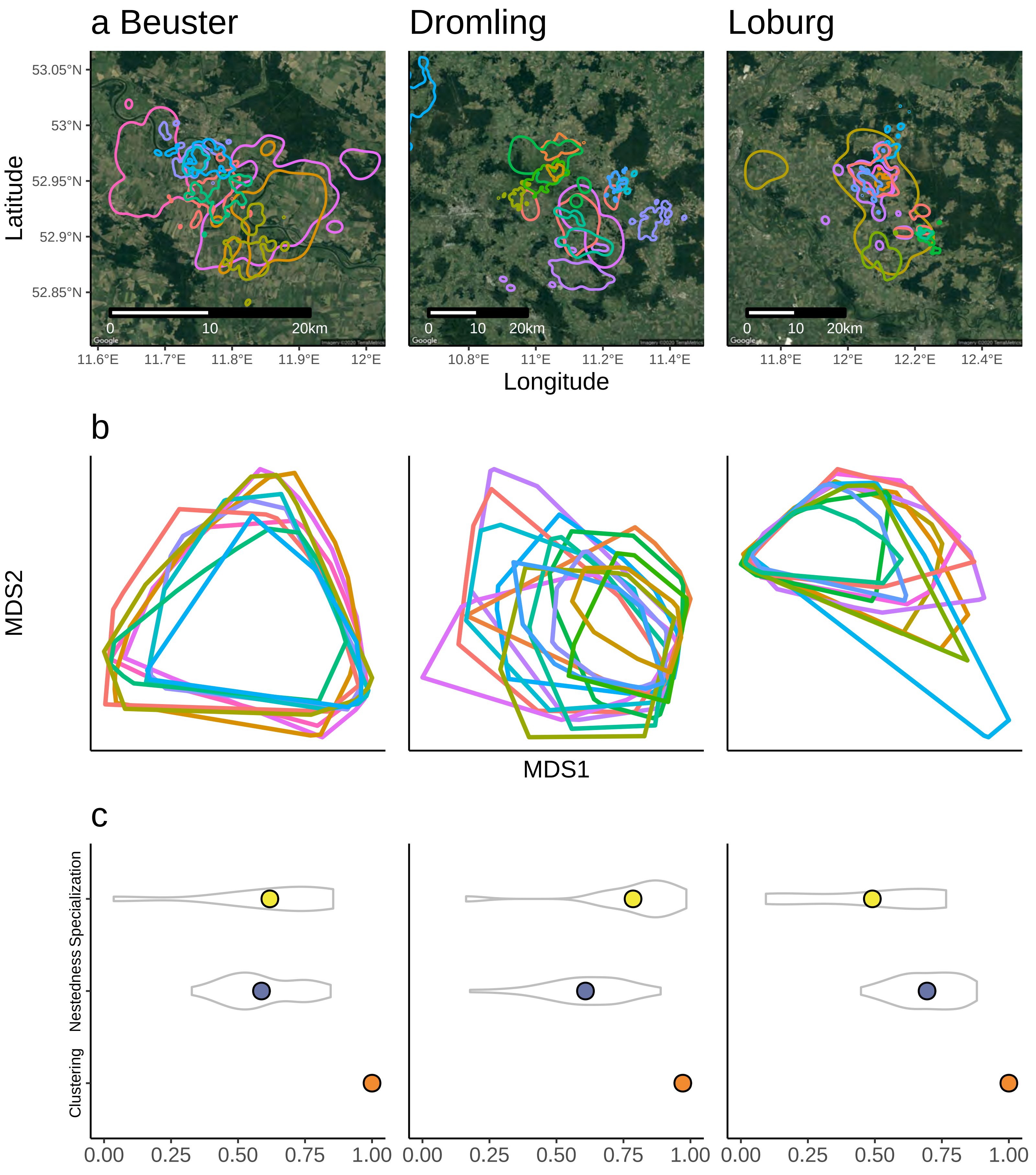
a Beuster
Dromling
Loburg
53.05°N
53°N
52.95°N
52.9°N
52.85°N
Latitude
0
10
20km
11.6°E
11.7°E
11.8°E
11.9°E
12°E
10.8°E
11°E
11.2°E
11.4°E
11.8°E
12°E
12.2°E
12.4°E
Longitude
b
MDS2
MDS1
c
Specialization
Nestedness
Clustering
0.00
0.25
0.50
0.75
1.00

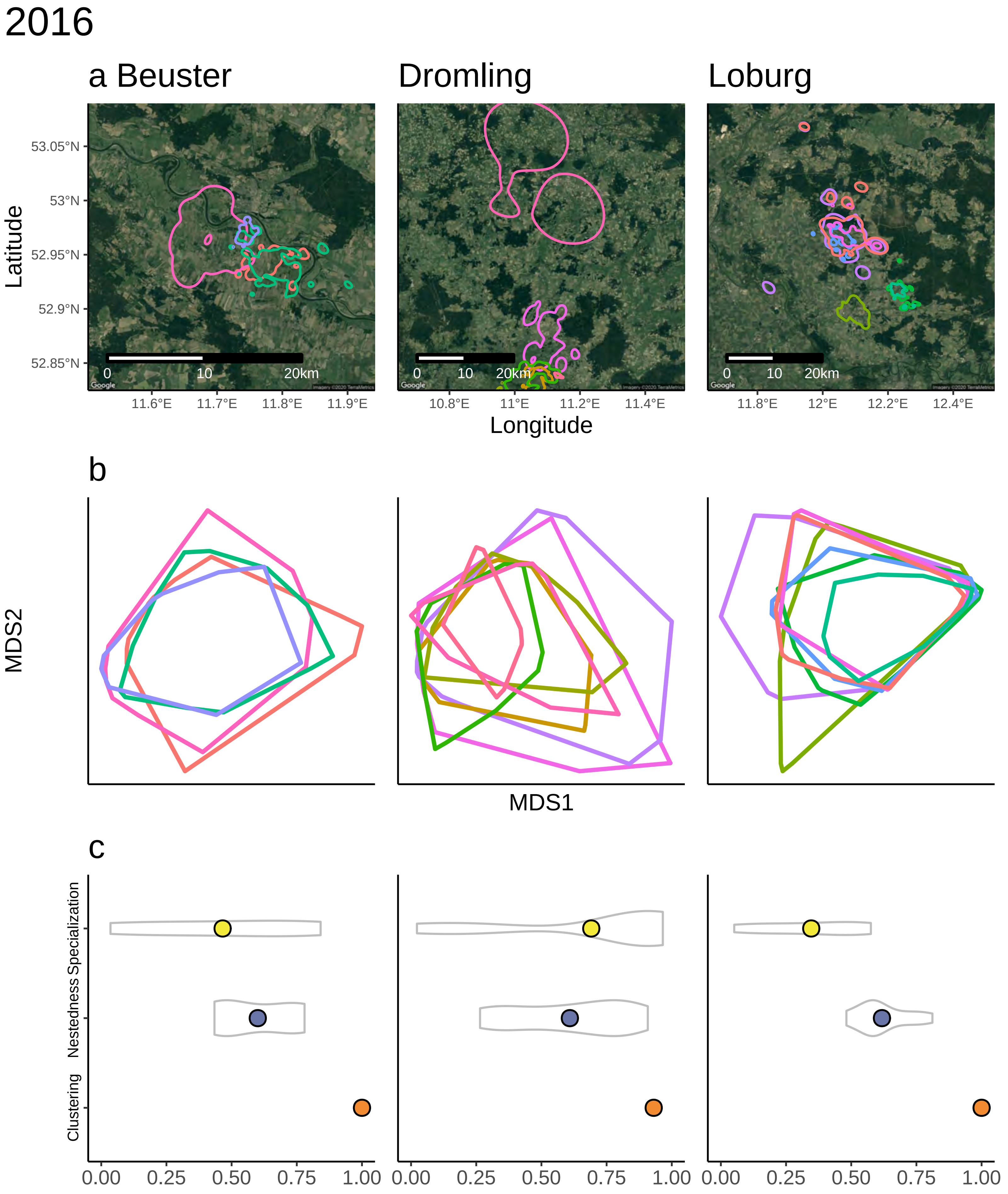
2016
a Beuster
Dromling
Loburg
Latitude
53.05°N
53°N
52.95°N
52.9°N
52.85°N
0
10
20km
11.6°E
11.7°E
11.8°E
11.9°E
10.8°E
11°E
11.2°E
11.4°E
11.8°E
12°E
12.2°E
12.4°E
Longitude
b
MDS2
MDS1
c
Nestedness Specialization
Clustering
0.00
0.25
0.50
0.75
1.00

Supplementary Figure 2. Home ranges (a), environmental niche configurations (b), and multivariate niche metrics (c) for three populations (Beuster, Drömling, Loburg) for three breeding seasons (2013, 2014, 2016). See Fig. 3 for 2015 breeding season and for further details. Basemap images in (a): Google, ©2020 TerraMetrics.

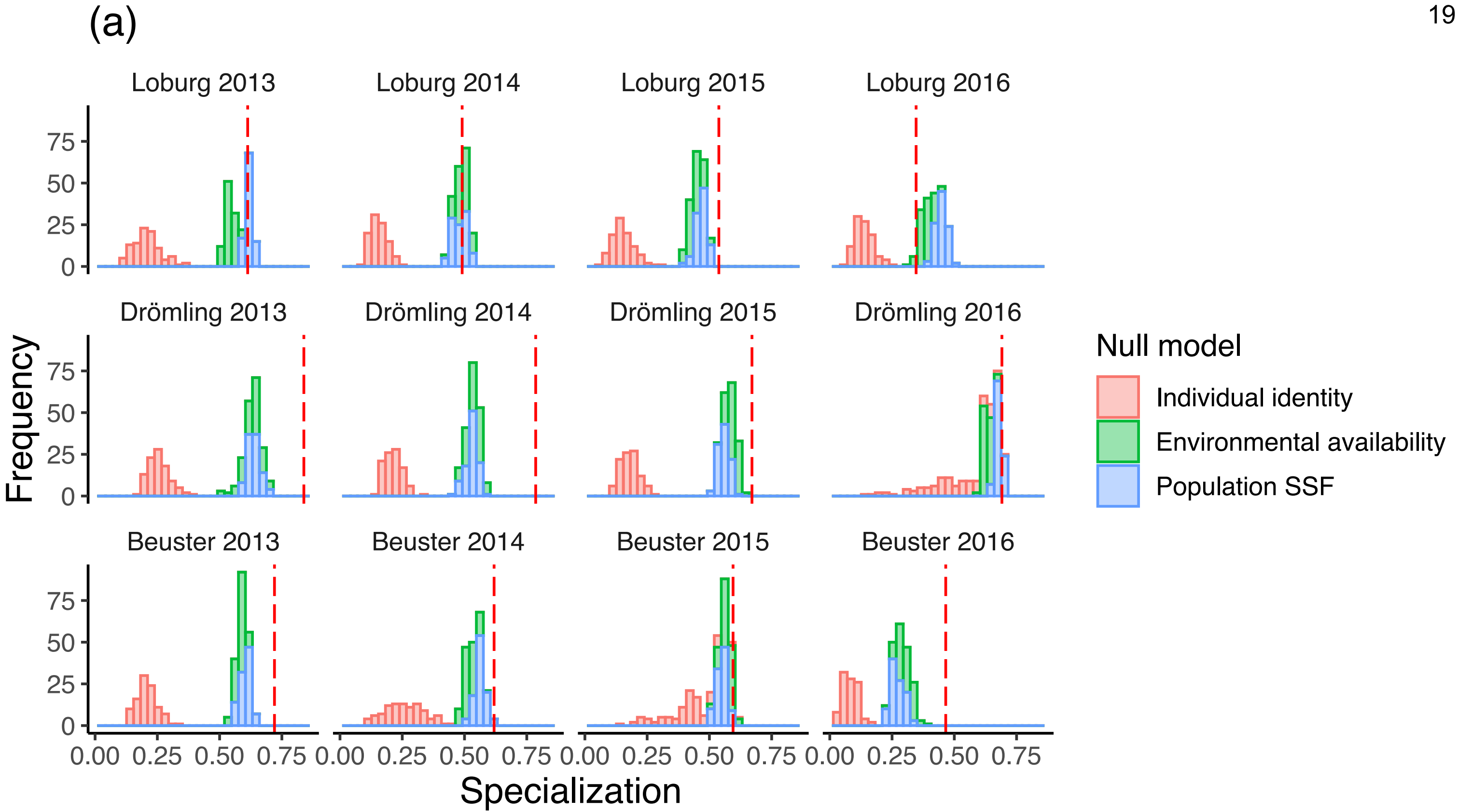
(a)
Loburg 2013
Loburg 2014
Loburg 2015
Loburg 2016
Drömling 2013
Drömling 2014
Drömling 2015
Drömling 2016
Beuster 2013
Beuster 2014
Beuster 2015
Beuster 2016
Frequency
0
25
50
75
0.00
0.25
0.50
0.75
Specialization
Null model
Individual identity
Environmental availability
Population SSF

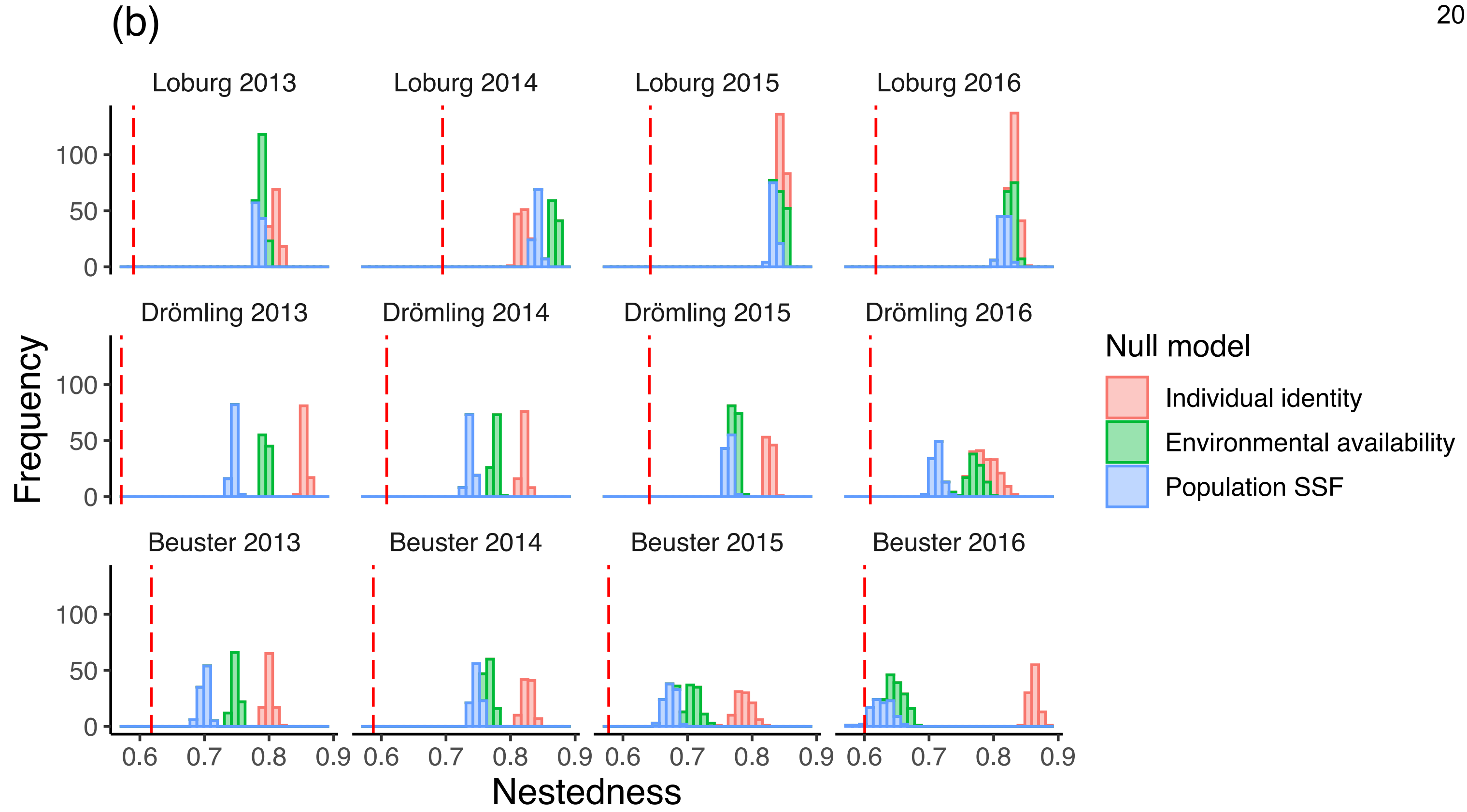
(b)
Loburg 2013
Loburg 2014
Loburg 2015
Loburg 2016
Drömling 2013
Drömling 2014
Drömling 2015
Drömling 2016
Beuster 2013
Beuster 2014
Beuster 2015
Beuster 2016
Frequency
0
50
100
0.6
0.7
0.8
0.9
Nestedness
Null model
Individual identity
Environmental availability
Population SSF

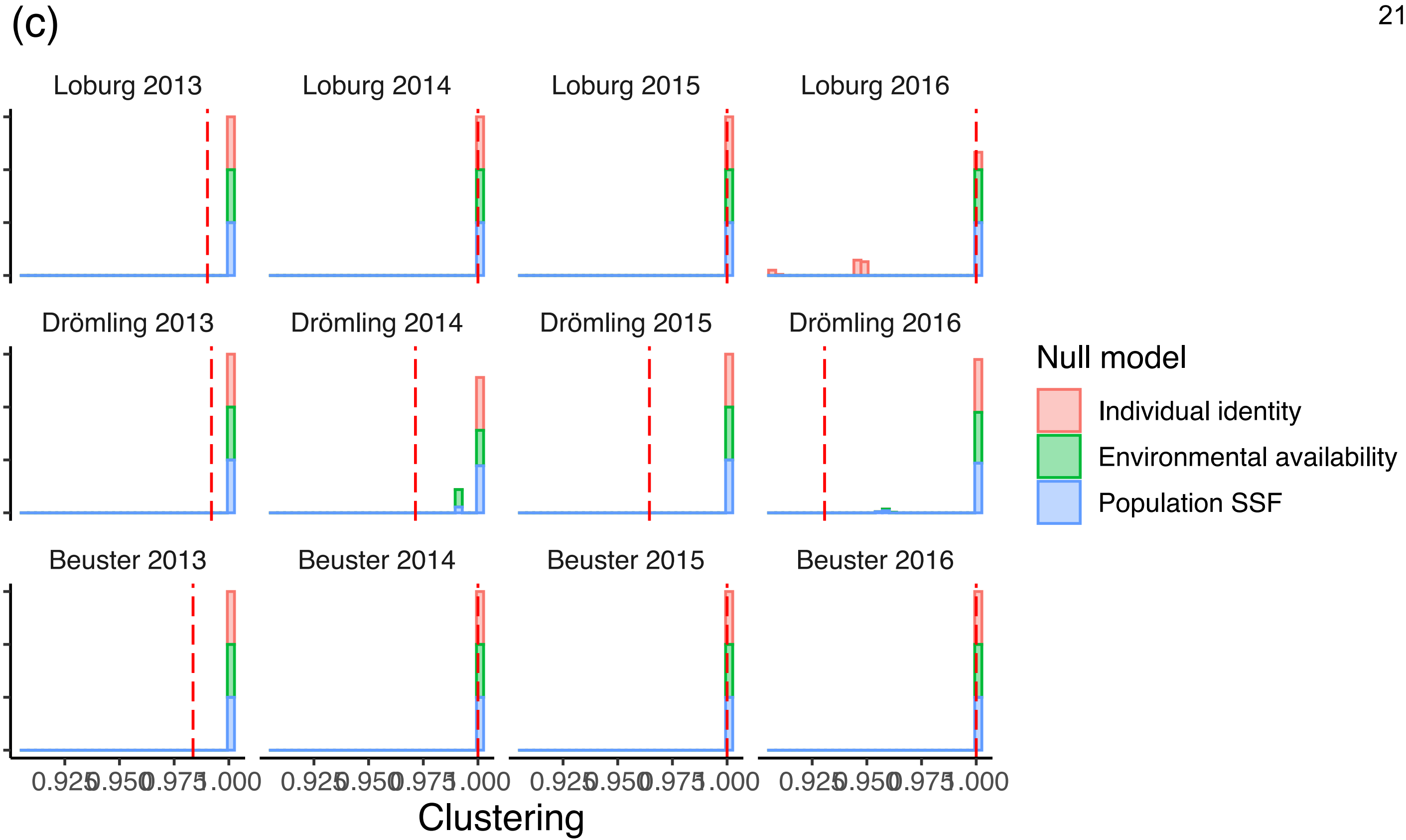
(c)
Loburg 2013
Loburg 2014
Loburg 2015
Loburg 2016
Drömling 2013
Drömling 2014
Drömling 2015
Drömling 2016
Beuster 2013
Beuster 2014
Beuster 2015
Beuster 2016
Frequency
0
100
200
300
Clustering
0.925
0.950
0.975
1.000
Null model
Individual identity
Environmental availability
Population SSF

Supplementary Figure 3. Distributions of (a) specialization, (b) nestedness, and (c) clustering metrics under three null models. Red dashed lines indicate the observed value of the niche metric. Individual identity: niches were randomly selected, with replacement, from the pooled observations within each population/year. The null model is that observed metrics are not driven by individual identity. Environmental availability: metrics are based on samples from environmental conditions available to each individual, and assumes that individuals sample the environment in proportion to availability. The null model is that observed metrics are due to differences in the available environment. Population SSF: the available distribution is the same as the null model for environmental availability, but individuals share the same SSF (a population-level SSF). The null model is that observed metrics are consistent with the same individual (the average stork) sampling the available environment. These analyses confirm that the level of specialization and nestedness is driven by individual identity assuming unrestricted availability ($p < .05$ for all population/years, for both specialization and nestedness), by individual environmental preferences assuming restricted availability and proportional sampling (specialization: $p < .05$ in ¾ of population/years, nestedness: $p < .05$ in all population/years), and by preferences assuming both restricted availability and a population-level SSF (specialization: $p < .05$ in ¾ of population/years, nestedness: $p < .05$ in 11/12 of population/years). Clustering in all cases had very little variation, was always near one, and was not different from the null distribution. The p-values represent two-sided, unadjusted values calculated from the bootstrapped null models as described above and in the methods section.

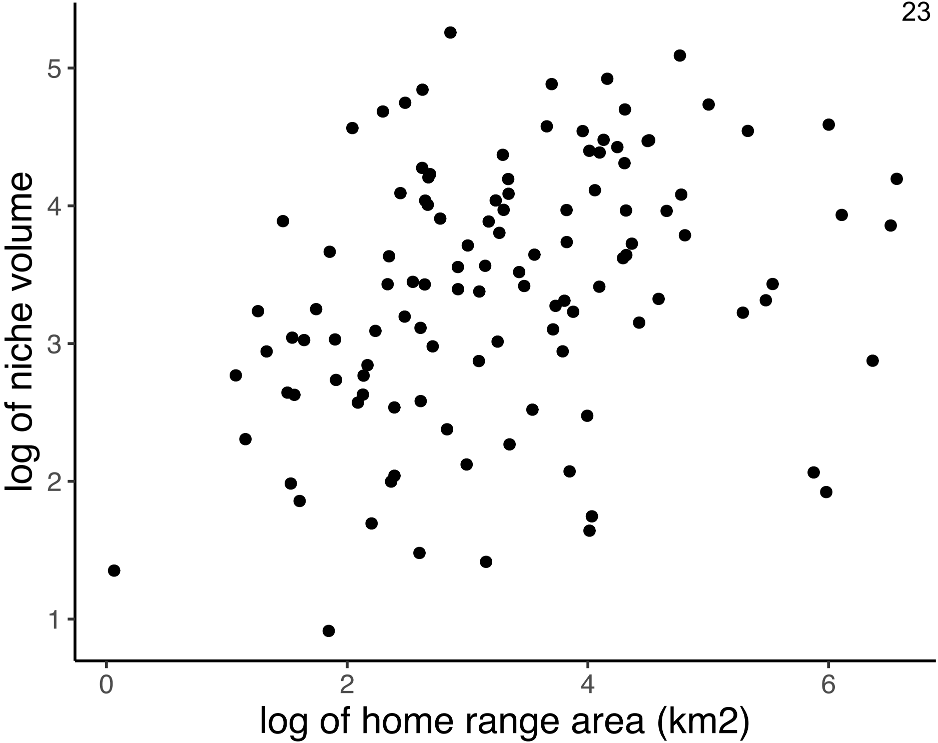
log of niche volume
1
2
3
4
5
0
2
4
6
log of home range area (km2)

Supplementary Figure 4. Scatter plot of home range area vs. niche volume. Although logarithms of these quantities show moderate correlation (r=0.3), home range area is not a strong driver of niche volume ($r^2 = 0.11$).

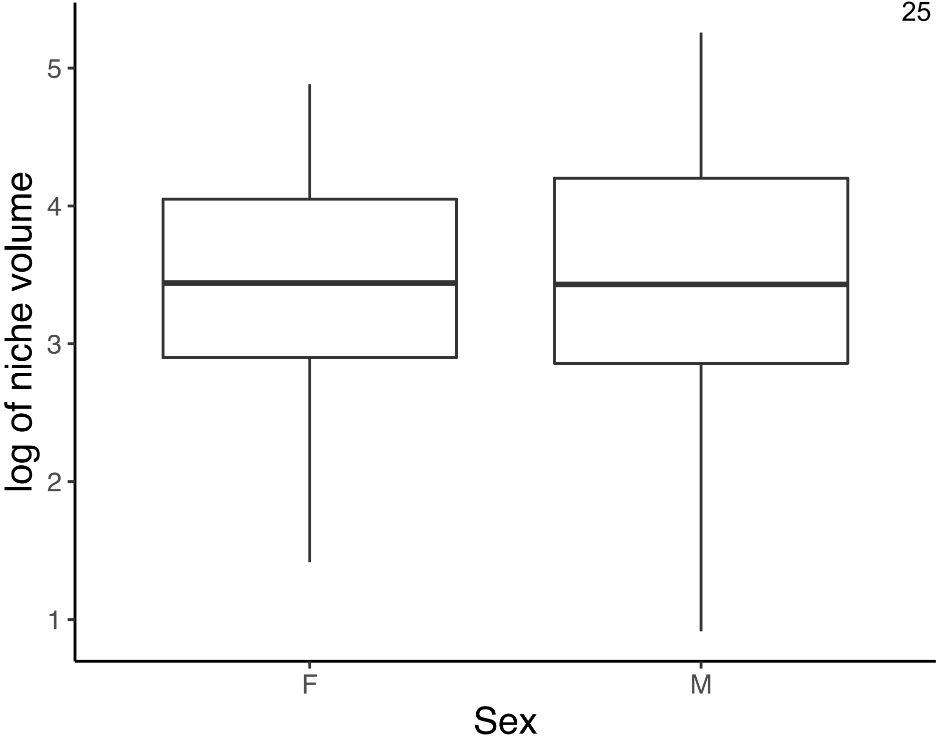

log of niche volume
5
4
3
2
1
F
M
Sex

Supplementary Figure 5. Boxplots of niche volume by sex. There appear to be no differences in niche volume due to sex. n=115 measurements of niche volume, from 44 individuals over up to four breeding seasons (one to four measurements per individual). Sex is unknown for one individual so it not included in this figure. The upper and lower hinges represent 25% and 75% quantiles, respectively. The middle line is the median. The upper whisker extends above the upper hinge and represents the greatest value less than 1.5 x IRQ above the hinge. The lower whisker extends below the lower hinge and represents the smallest value greater than 1.5 x IRQ below the hinge. IRQ is the interquartile range.

Distance to forest

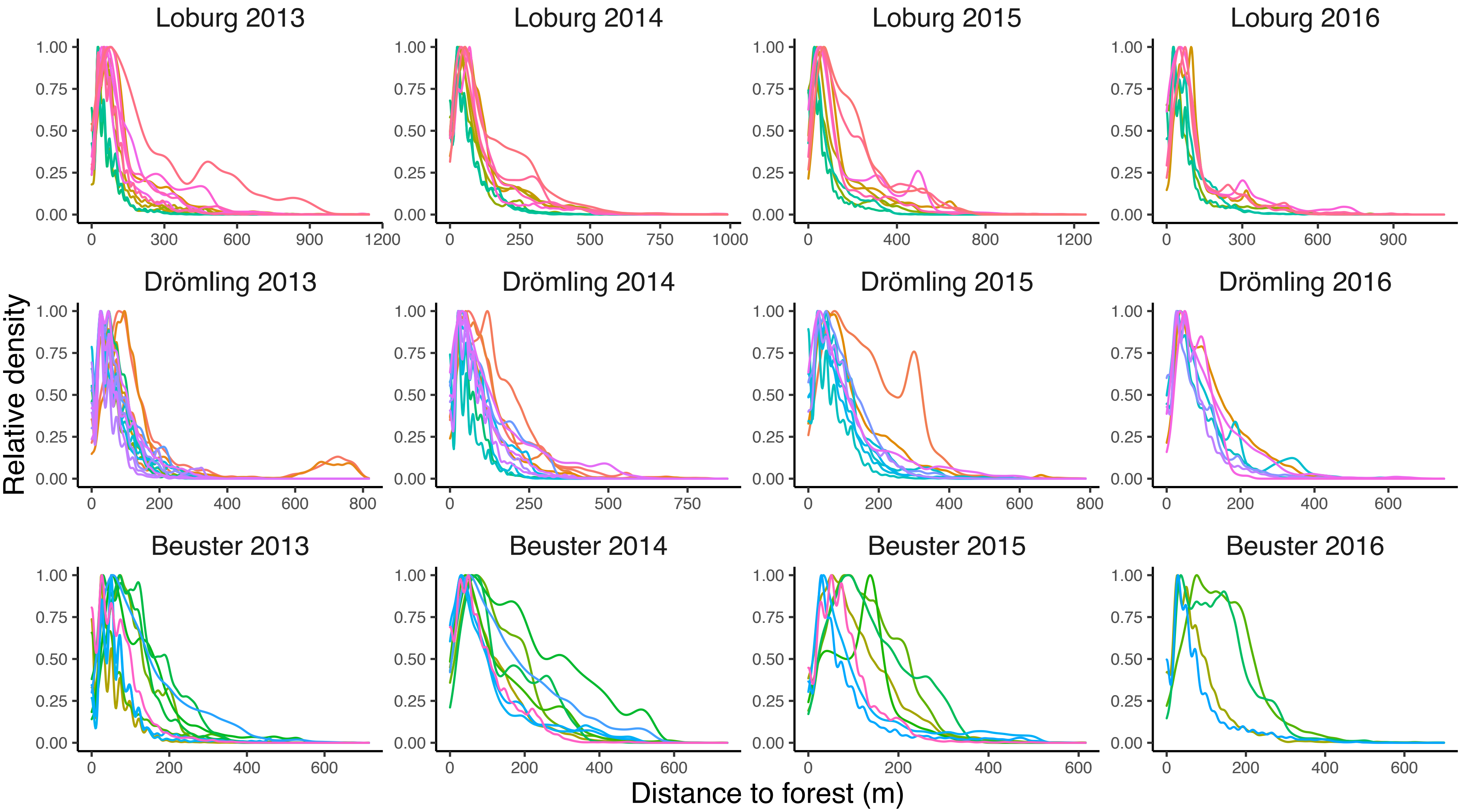

Distance to urban

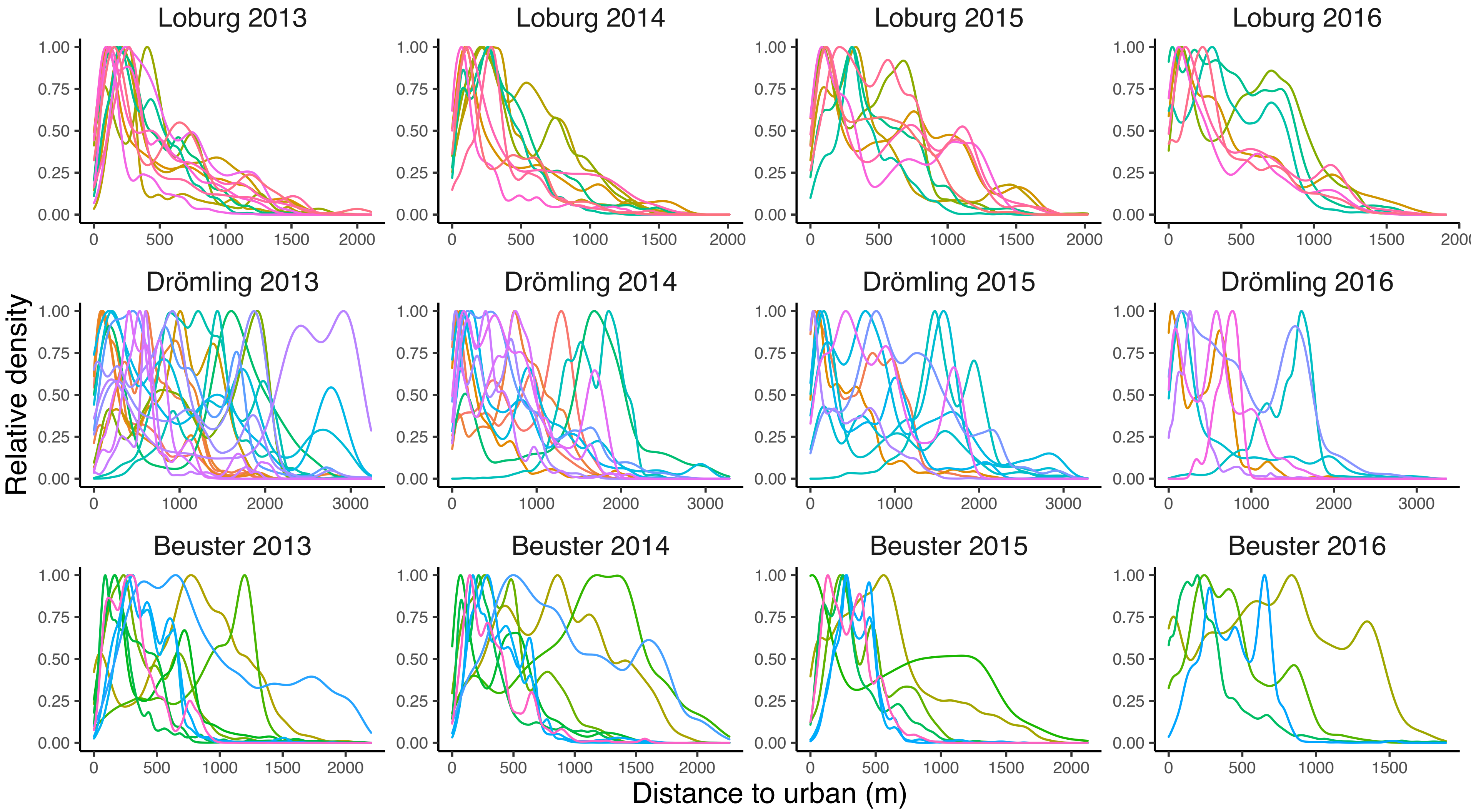

NDVI (16-day)

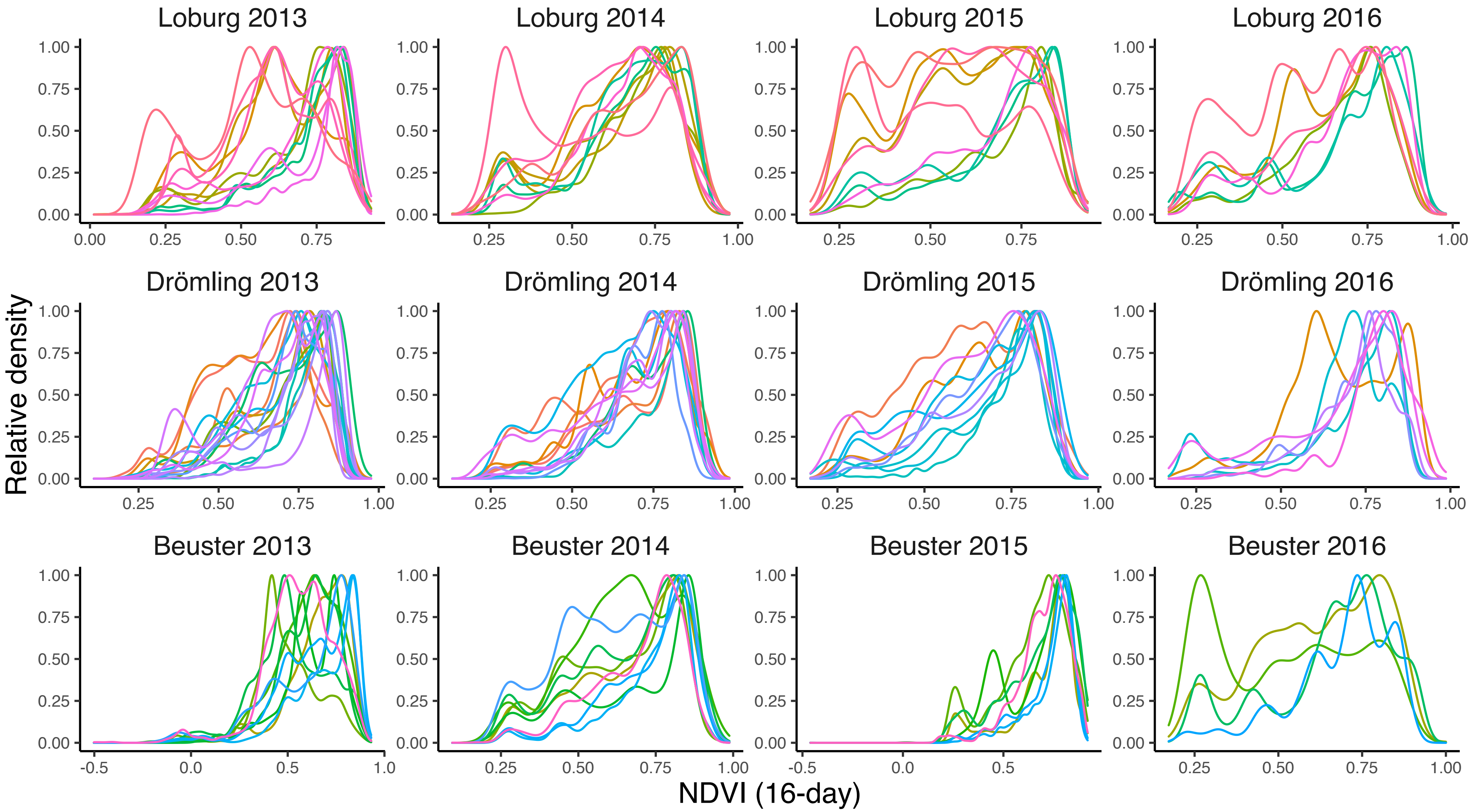
Loburg 2013
Loburg 2014
Loburg 2015
Loburg 2016
Drömling 2013
Drömling 2014
Drömling 2015
Drömling 2016
Beuster 2013
Beuster 2014
Beuster 2015
Beuster 2016
Relative density
NDVI (16-day)

Percent bare

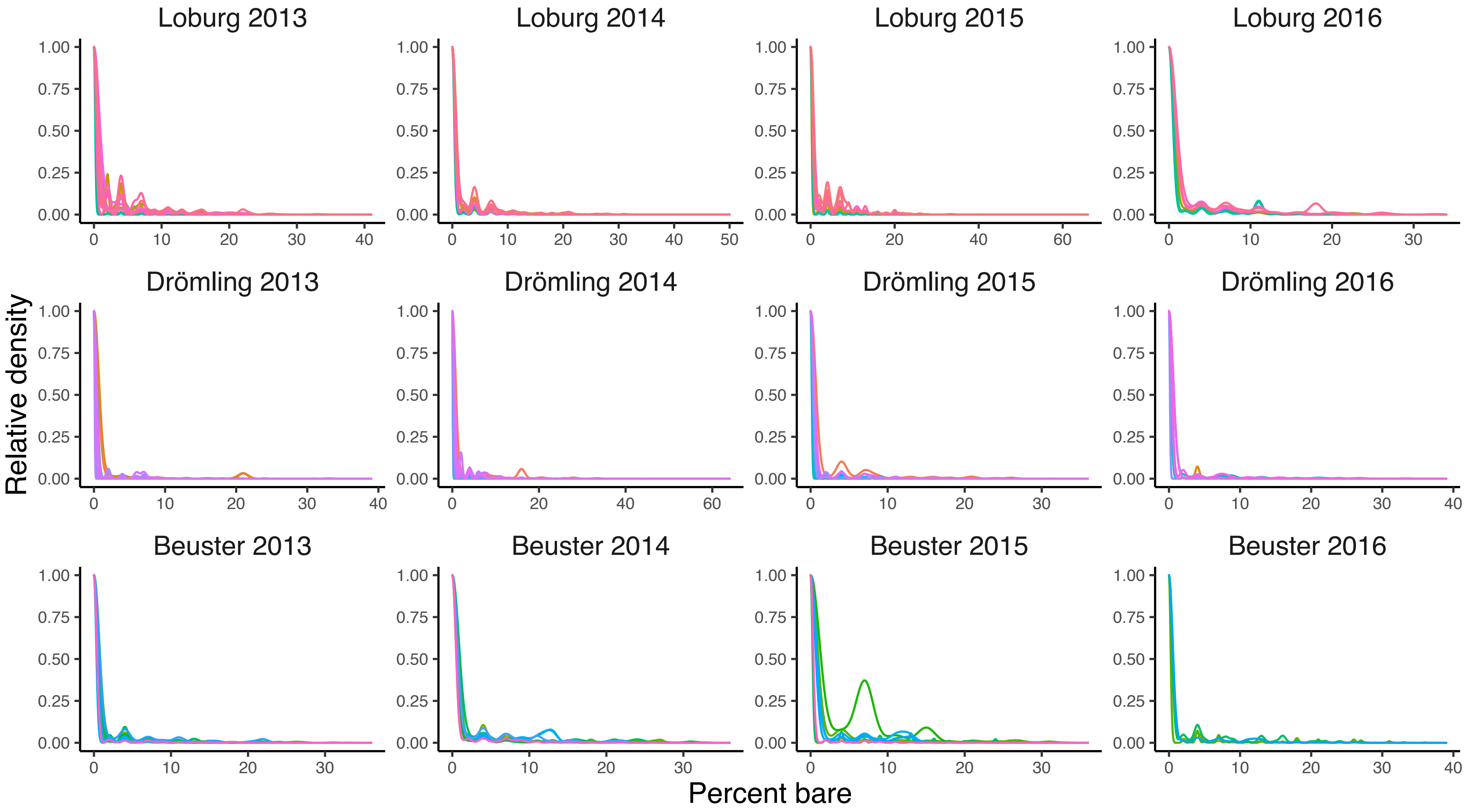

Percent tree

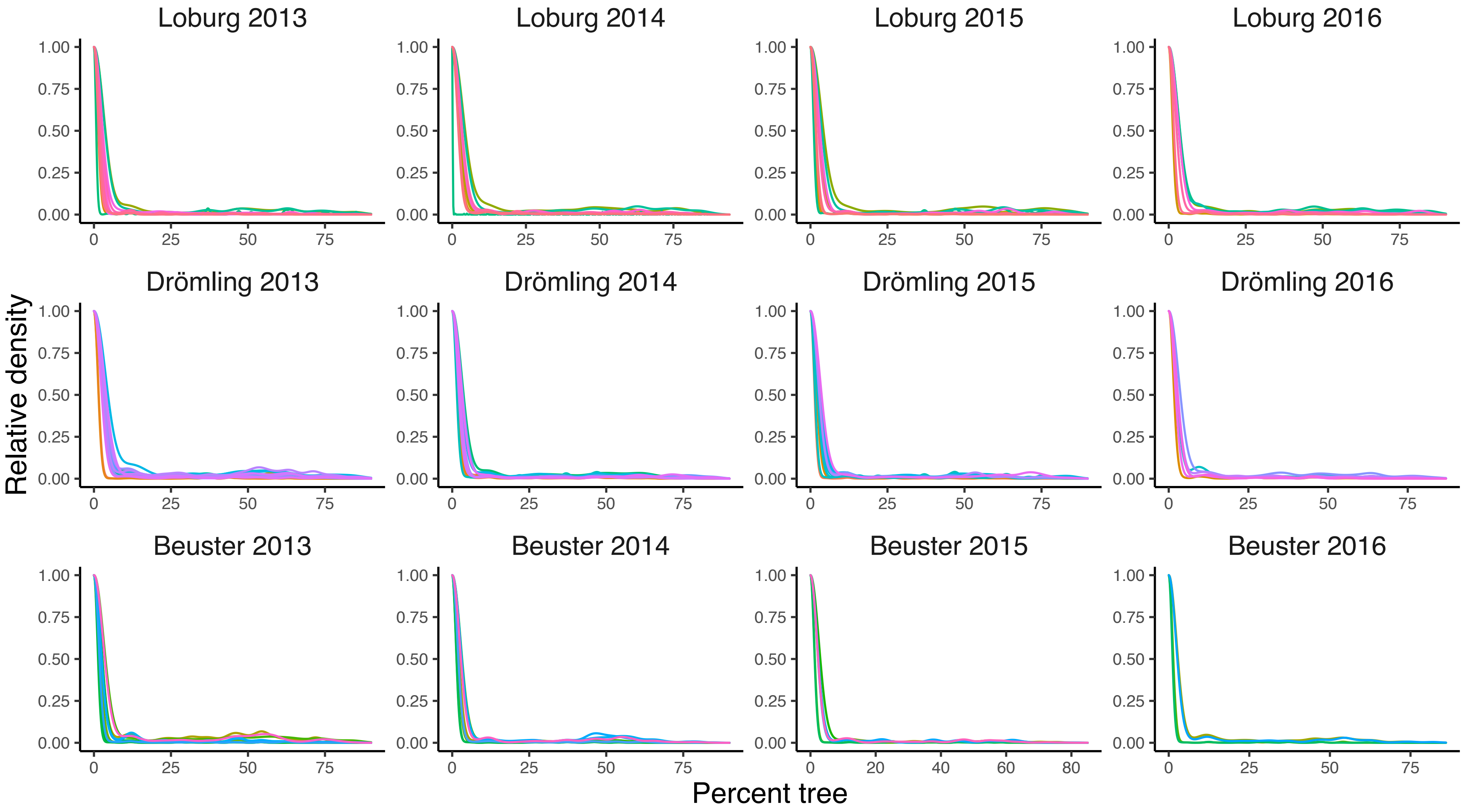

Supplementary Figure 6. Plots of relative density of habitat use for environmental variables defined in Table S1, for each population, year, and individual. Colors indicate different individuals.

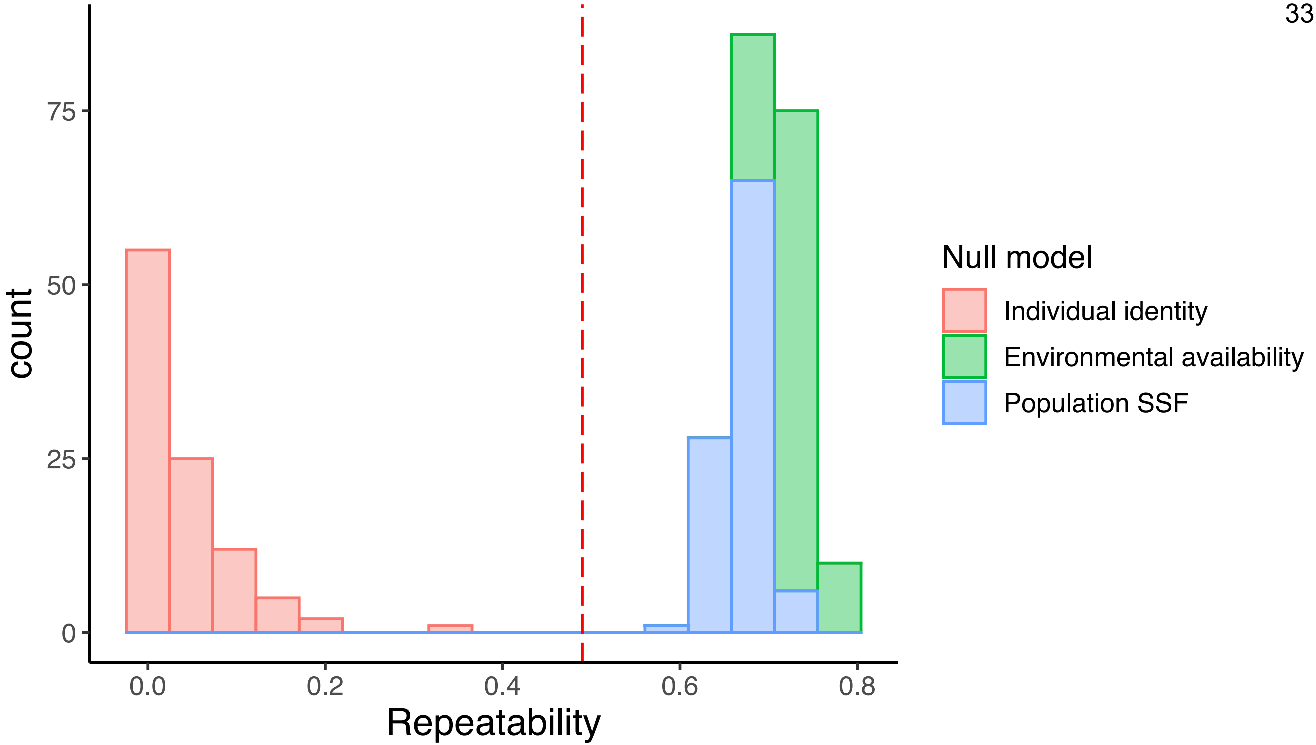

count
75
50
25
0
0.0
0.2
0.4
0.6
0.8
Repeatability
Null model
Individual identity
Environmental availability
Population SSF

Supplementary Figure 7. Distributions of repeatability under the three null models described in Fig. S3 and the methods section. The dashed line shows the observed repeatability. Under the null model for individual identity, we expect that repeatability will be lower because randomization of the individual identities results in lower among-individual variance. Under the null model for environmental availability, as well as the null model for population SSF, we expect that repeatability will be higher due to lower within-individual variance. This is because we sample according to environmental availability or a population-level (mean) SSF, respectively, both of which result in lower within-site variance over time. In all null models the observed value rejects the null hypothesis ($p < .01$).

Distance to forest

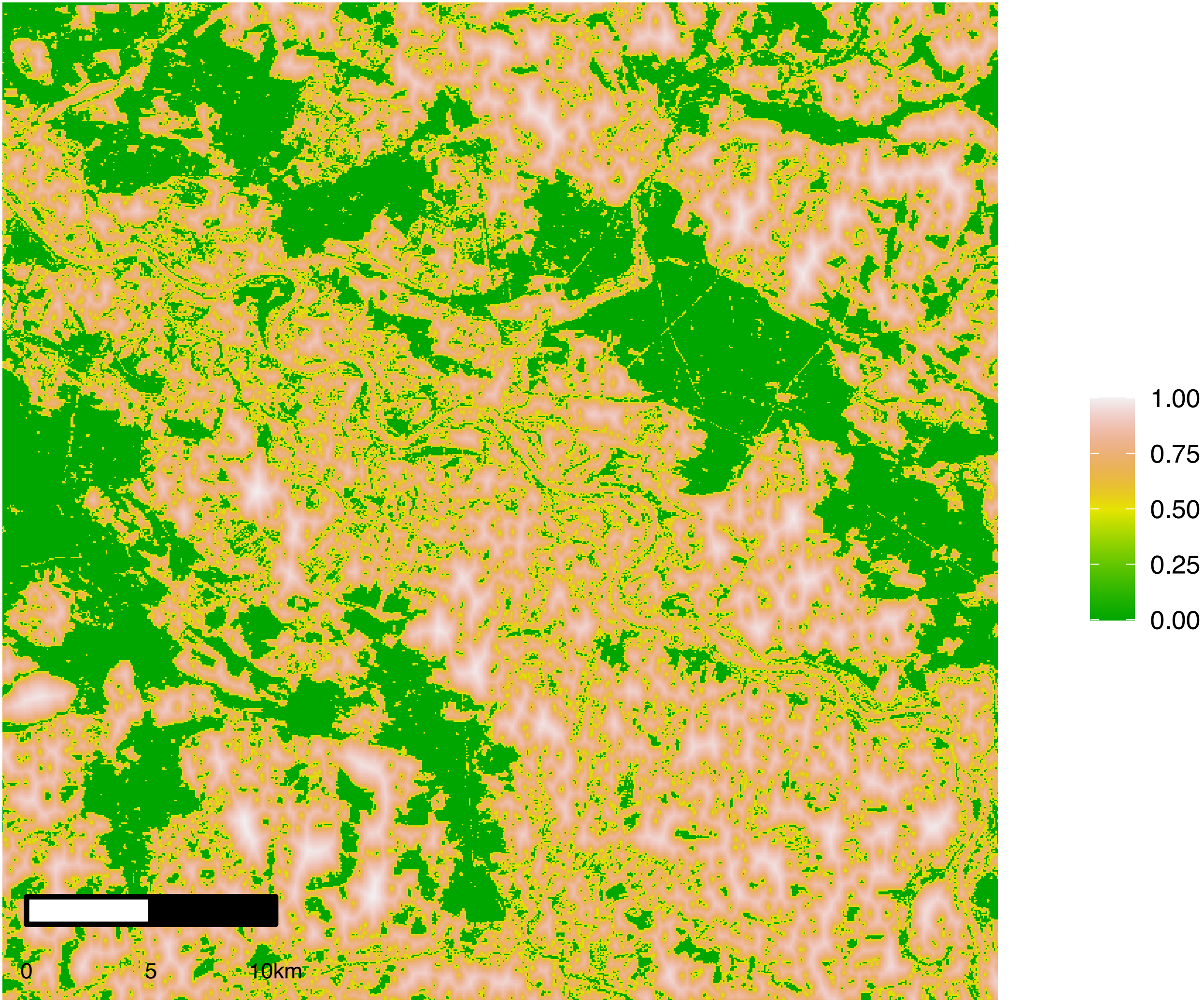

A log stretch has been applied to this layer.

Distance to urban

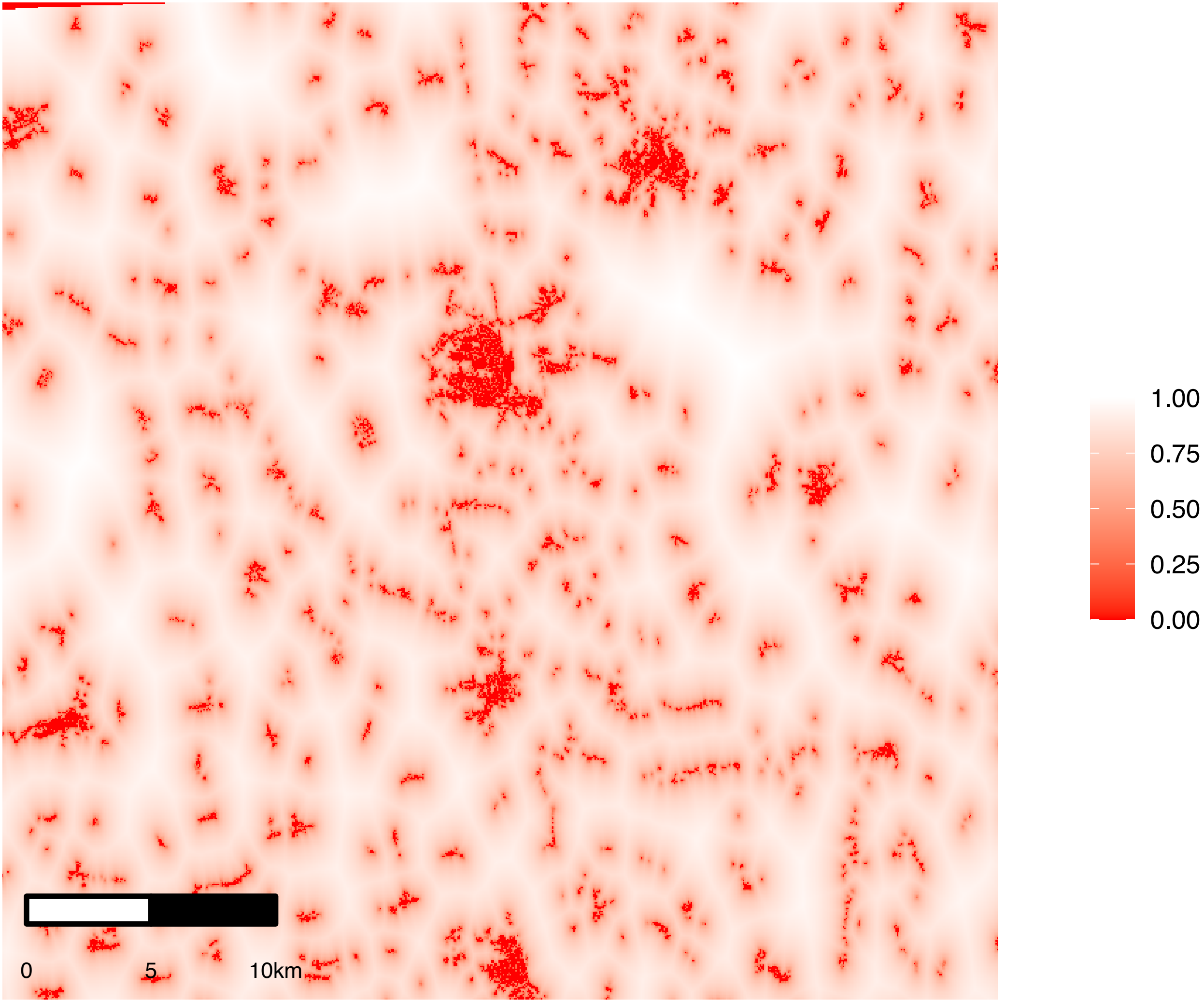

A log stretch has been applied to this layer.

Mean NDVI

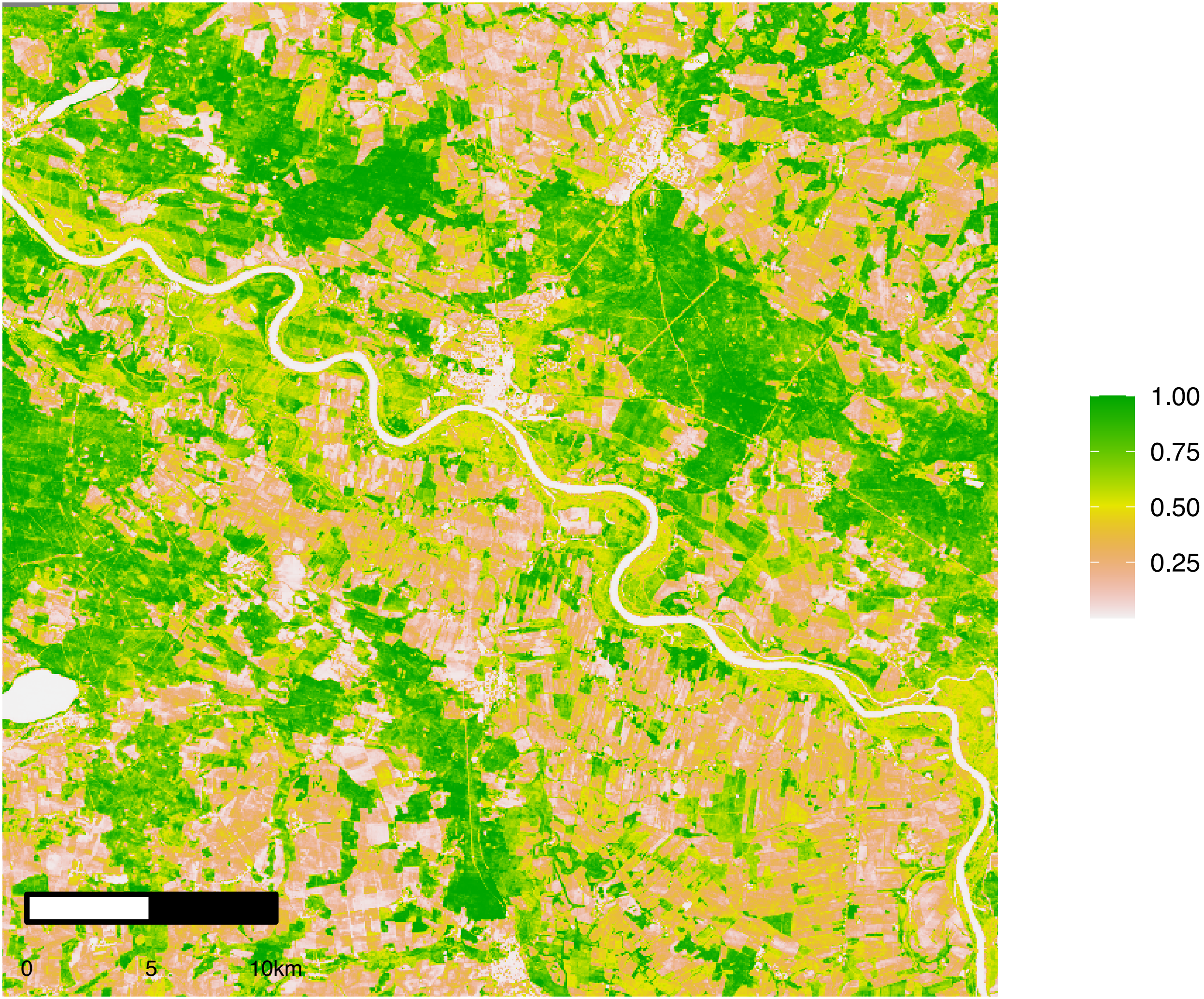

A histogram stretch has been applied to this layer.

CV of NDVI

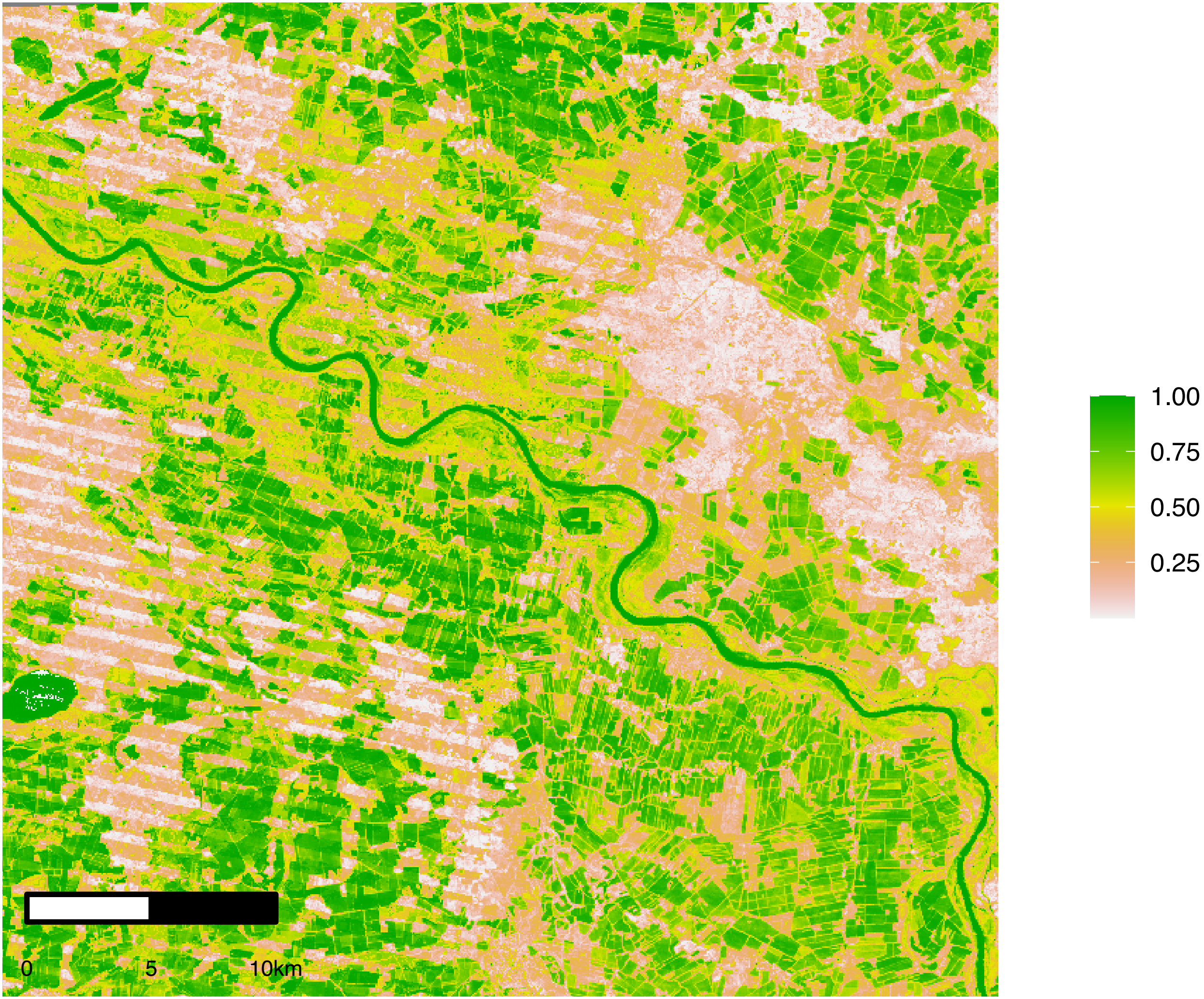

A histogram stretch has been applied to this layer.

Percent bare

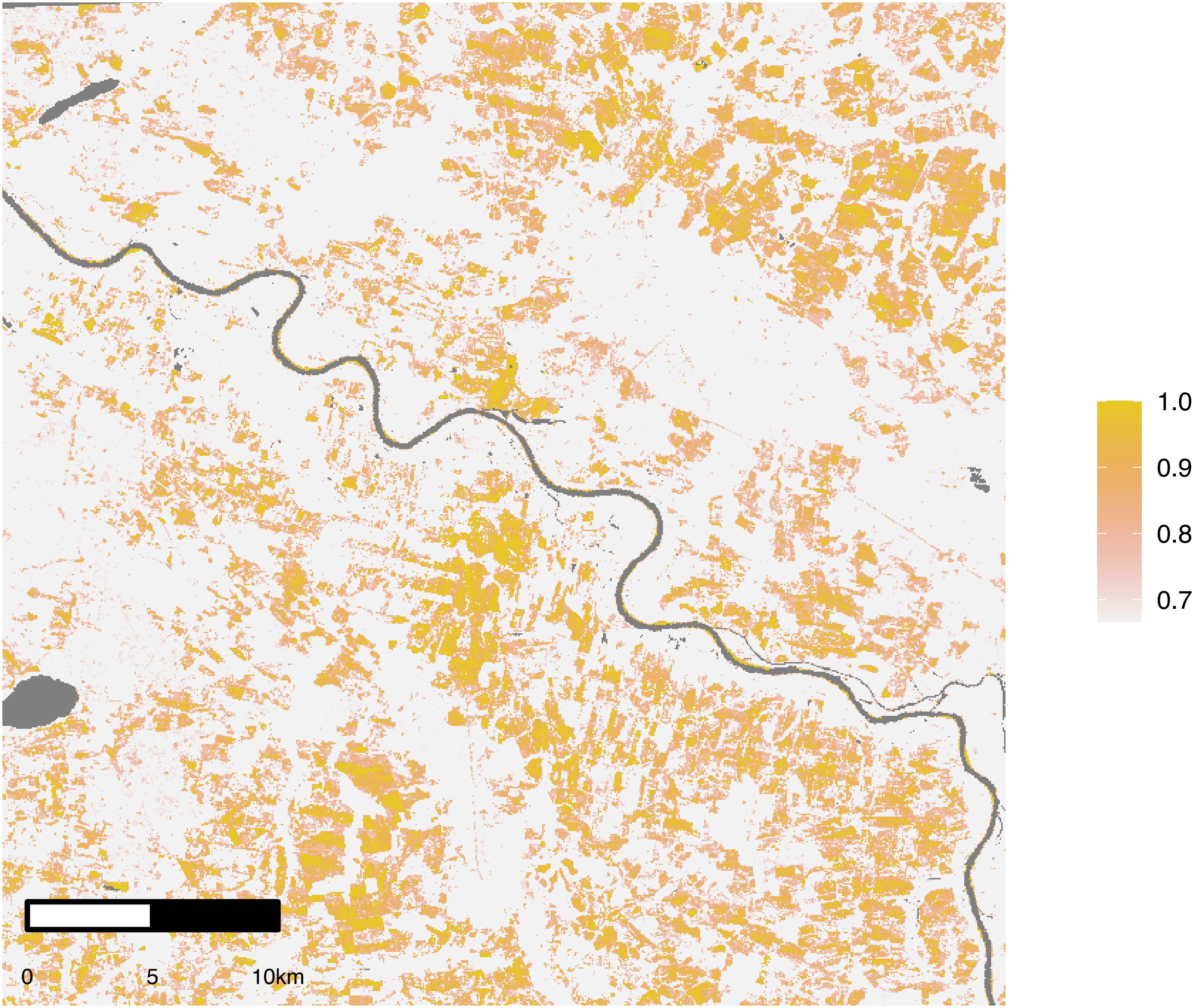

A histogram stretch has been applied to this layer.

Percent Tree

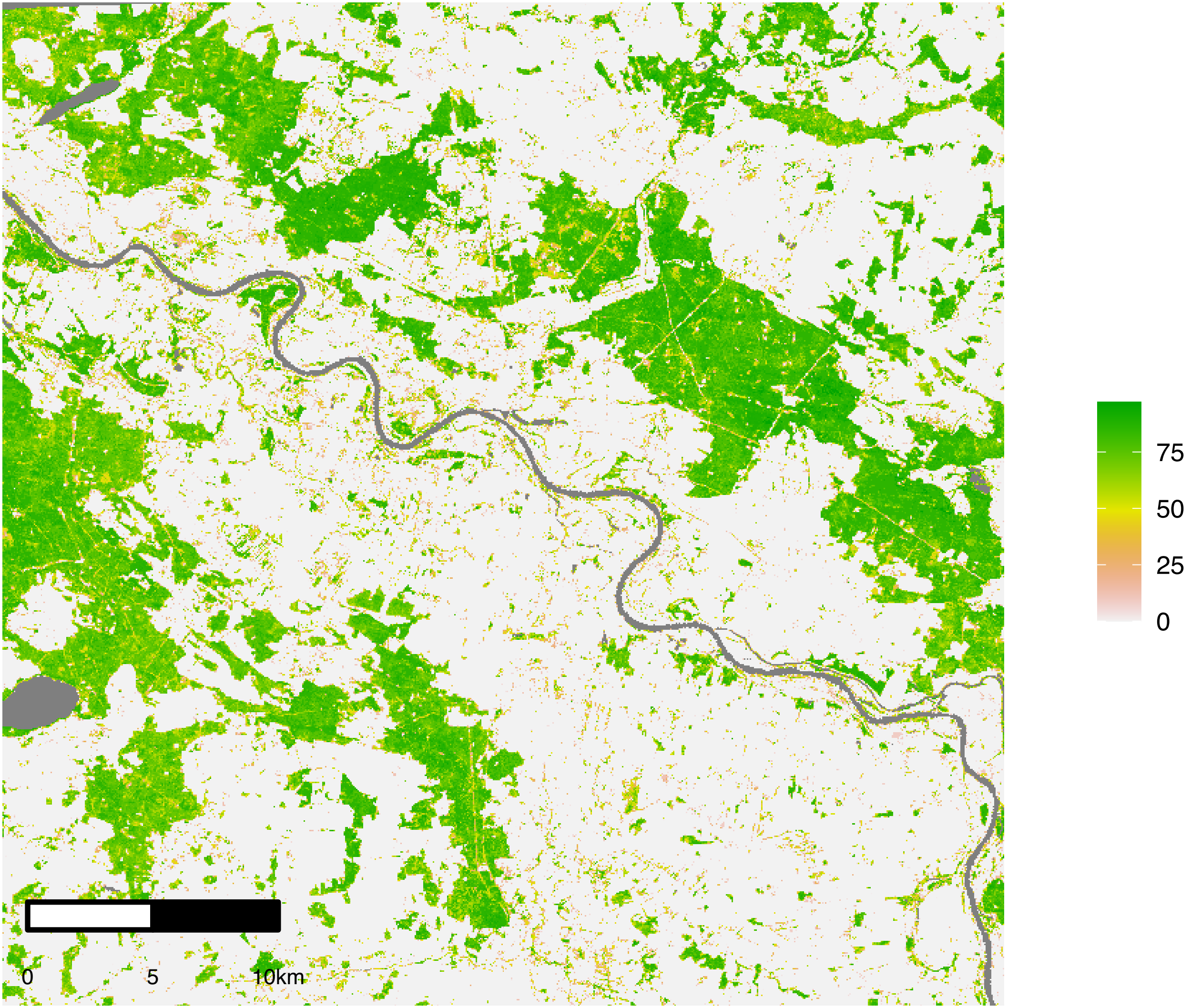

Supplementary Figure 8. Maps of the five environmental variables used in niche estimation for the Beuster population. Please see Table S1 for information about these variables. The NDVI variable is a time series (16-day), so mean and CV is presented.

Distance to forest

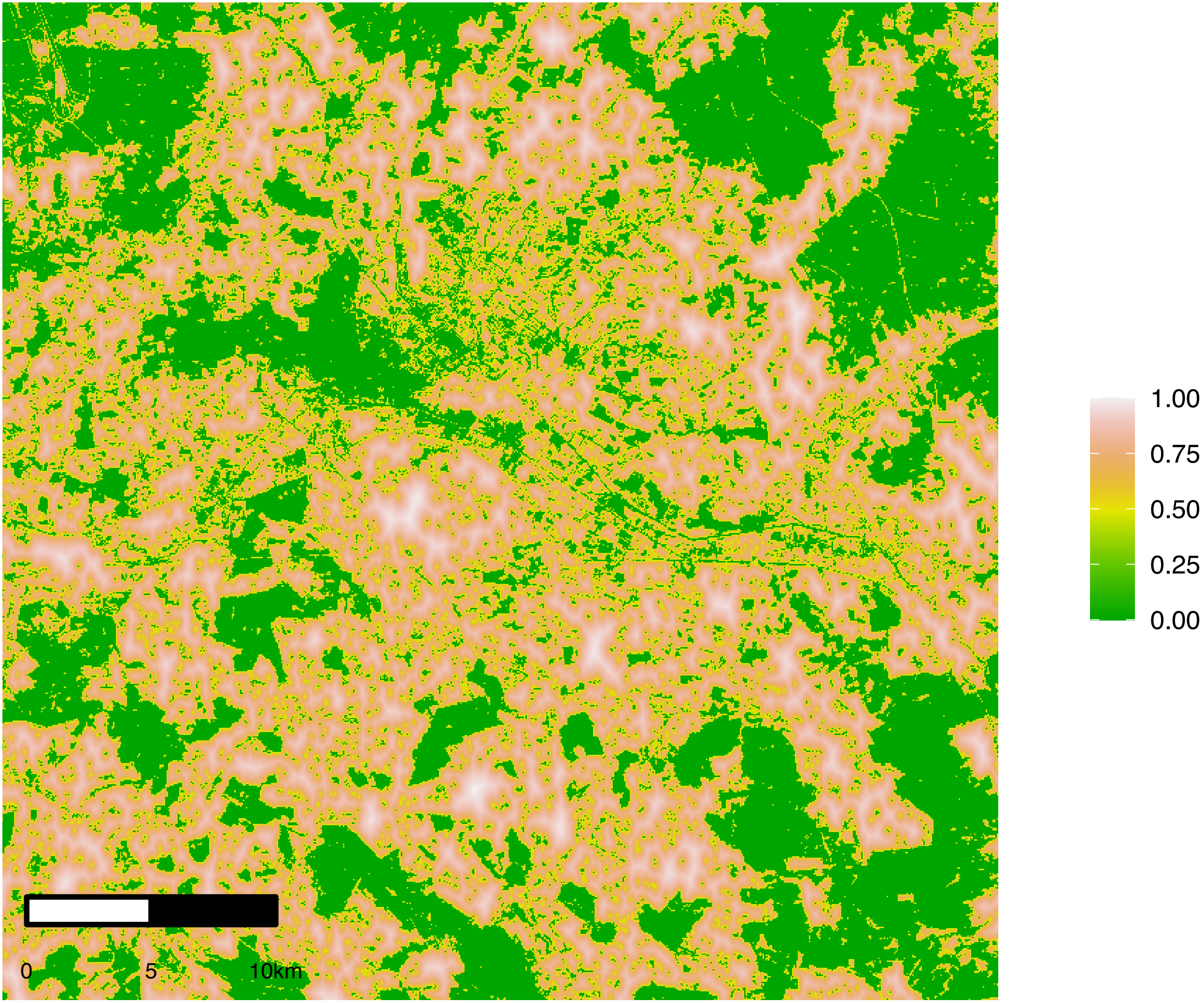

A log stretch has been applied to this layer.

Distance to urban

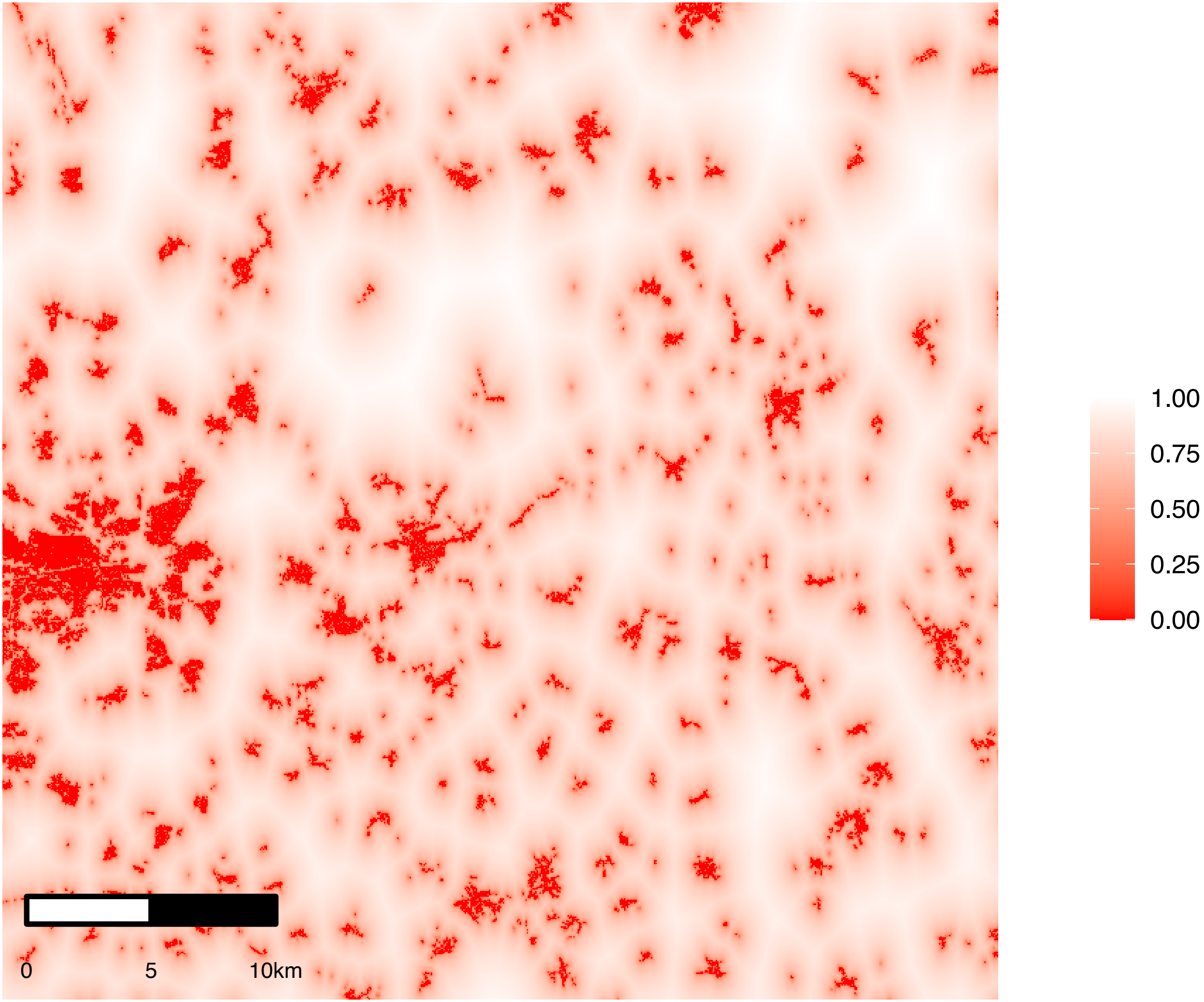

A log stretch has been applied to this layer.

Mean NDVI

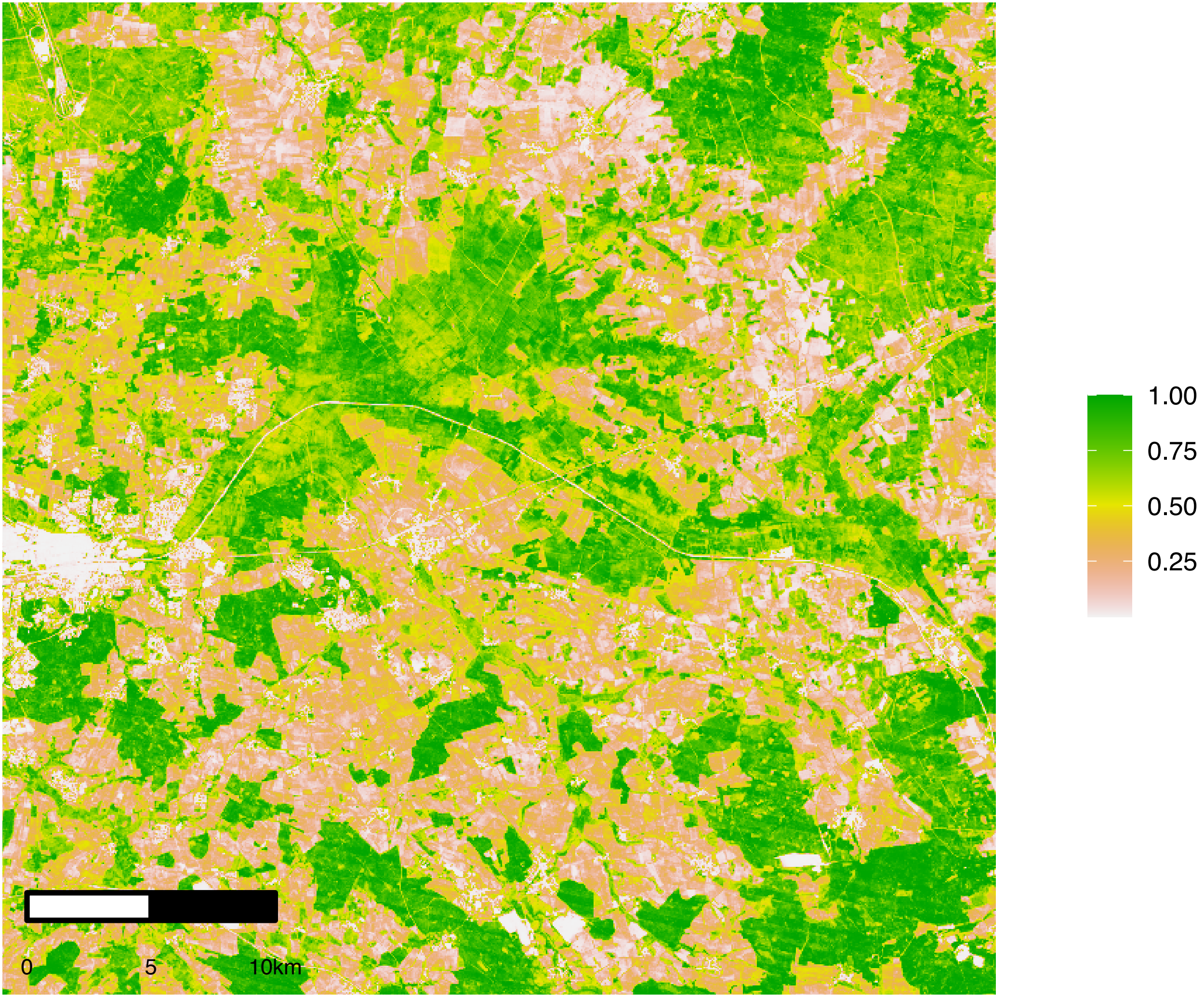

A histogram stretch has been applied to this layer.

CV of NDVI

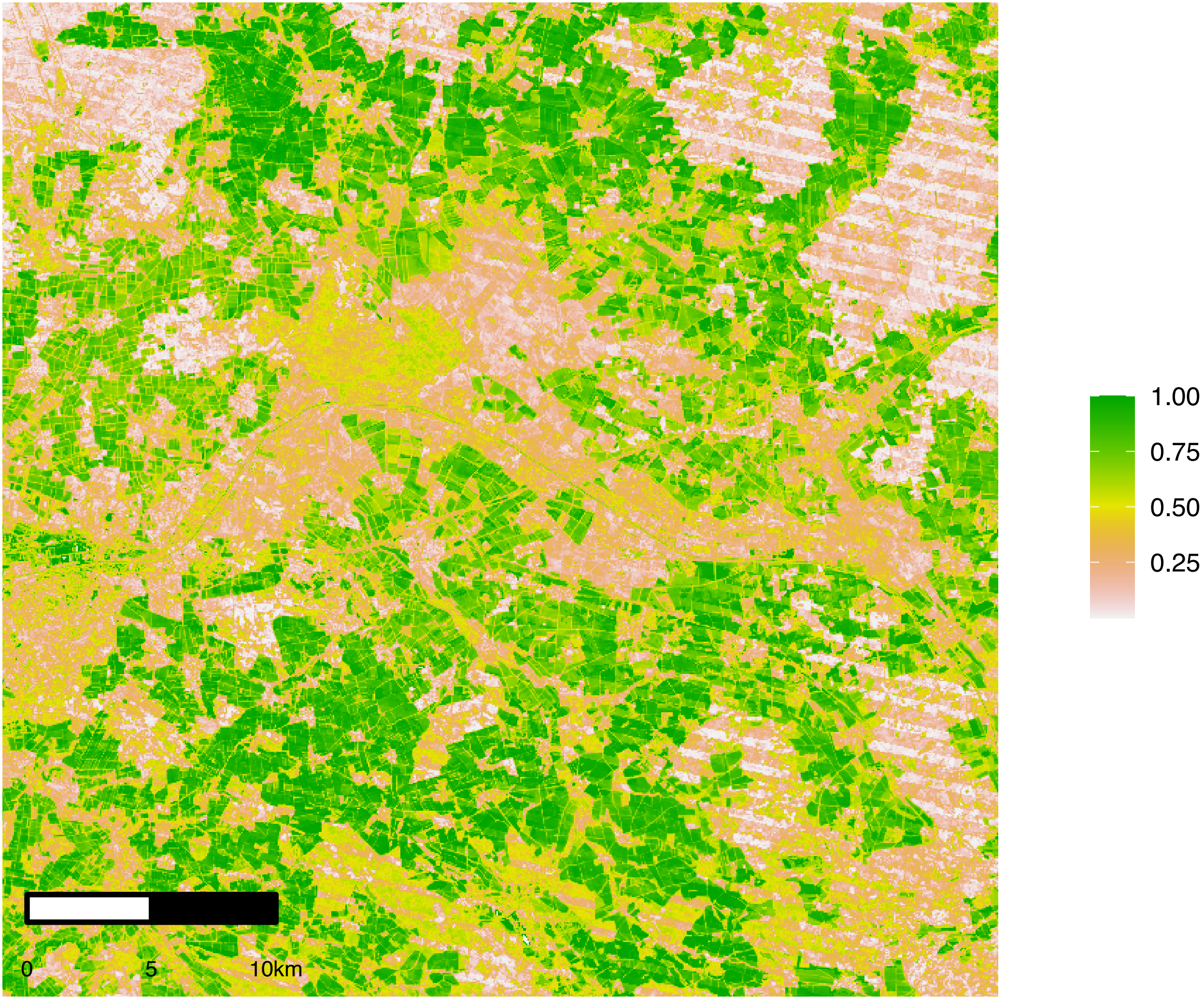

A histogram stretch has been applied to this layer.

Percent bare

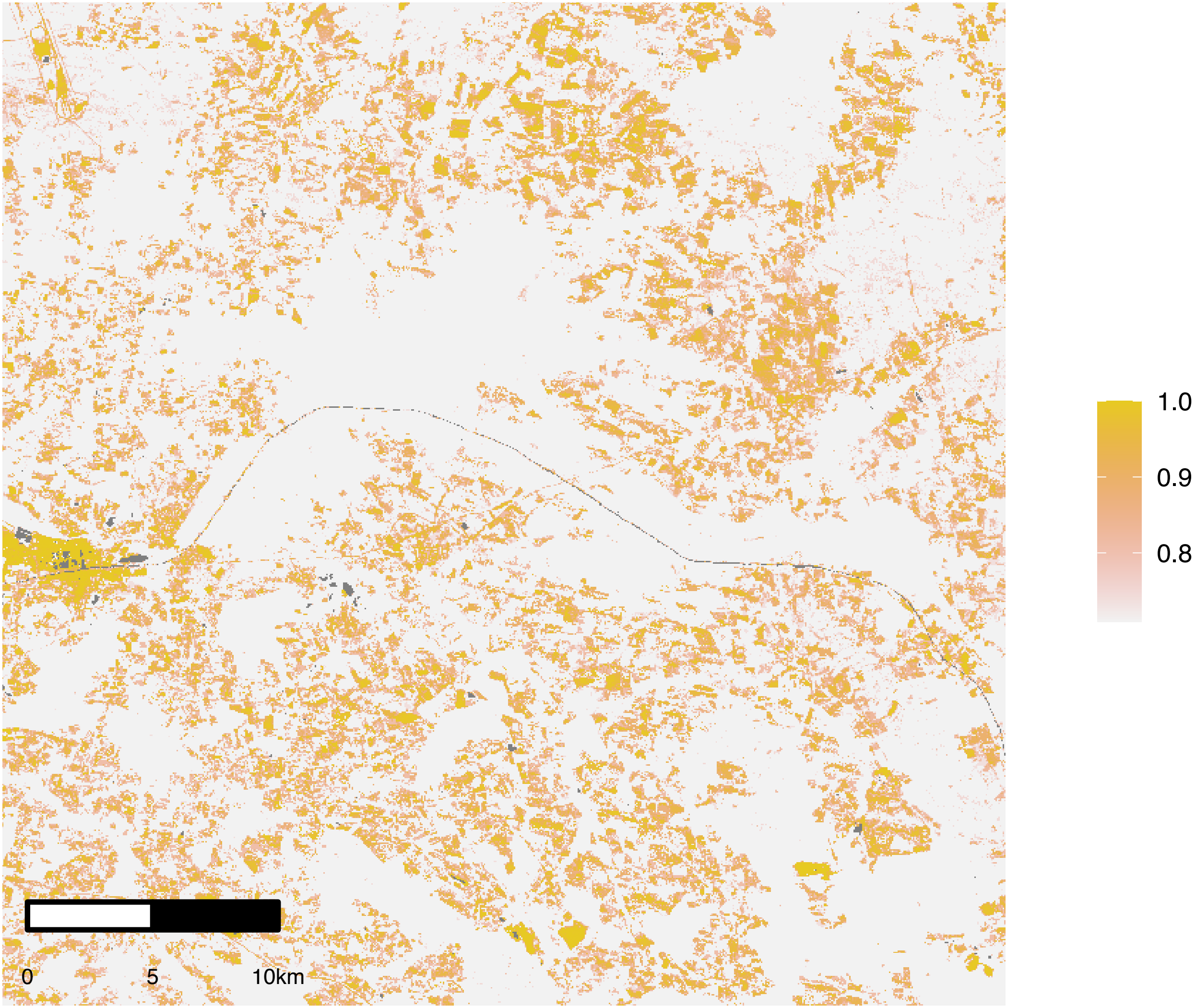

A histogram stretch has been applied to this layer.

Percent Tree

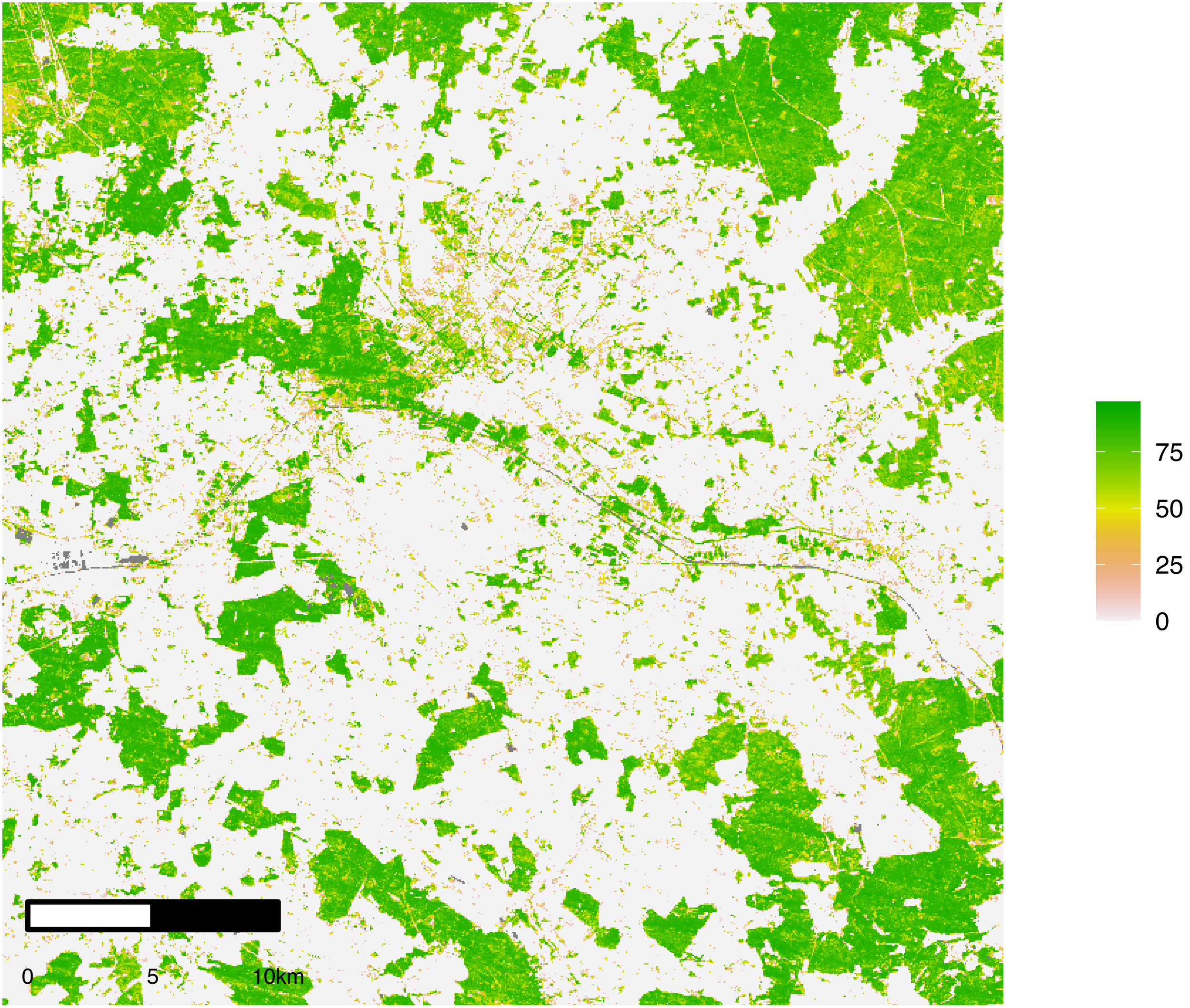

Supplementary Figure 9. Maps of the five environmental variables used in niche estimation for the Drömling population. Please see Table S1 for information about these variables. The NDVI variable is a time series (16-day), so mean and CV is presented.

Distance to forest

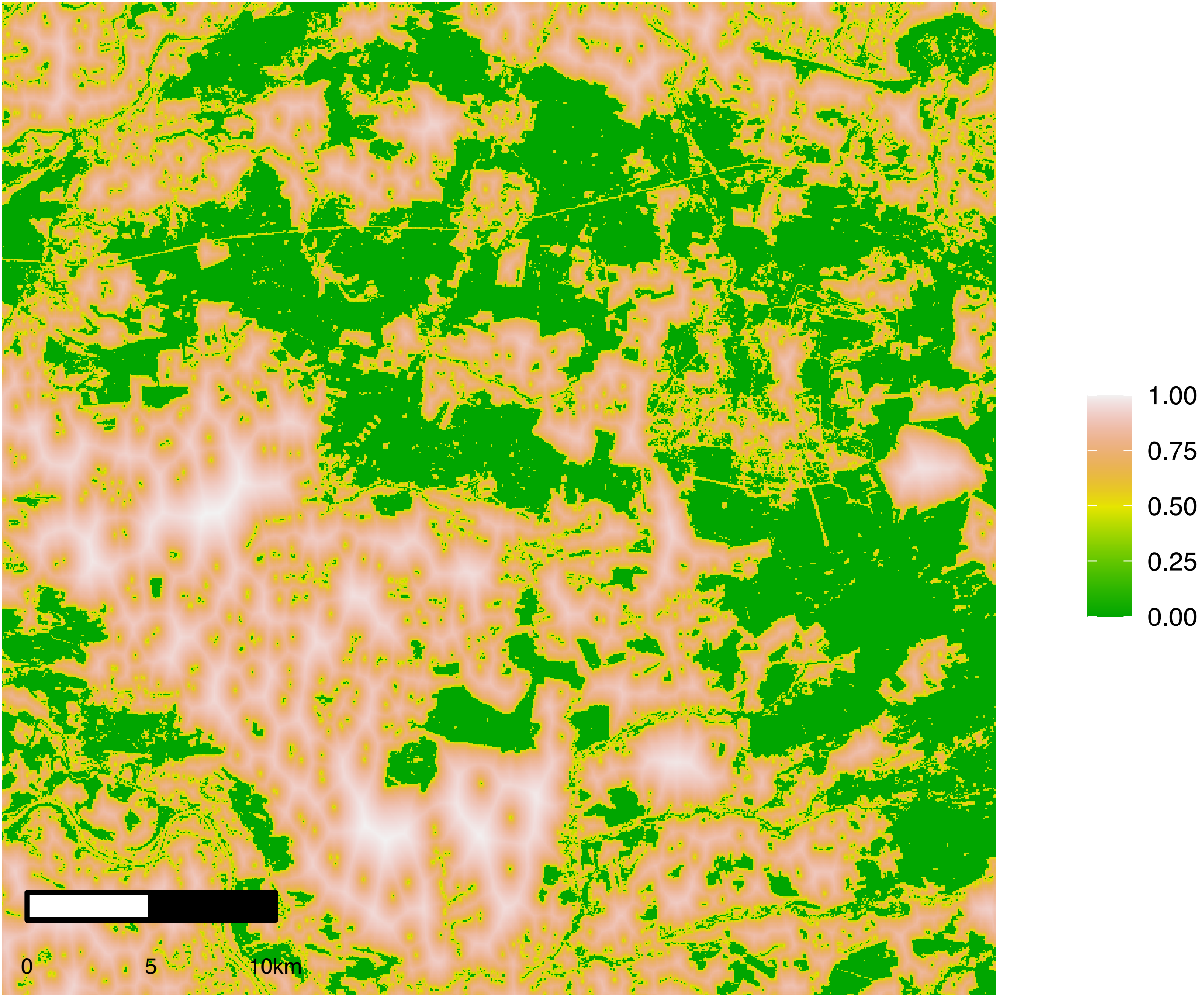

A log stretch has been applied to this layer.

Distance to urban

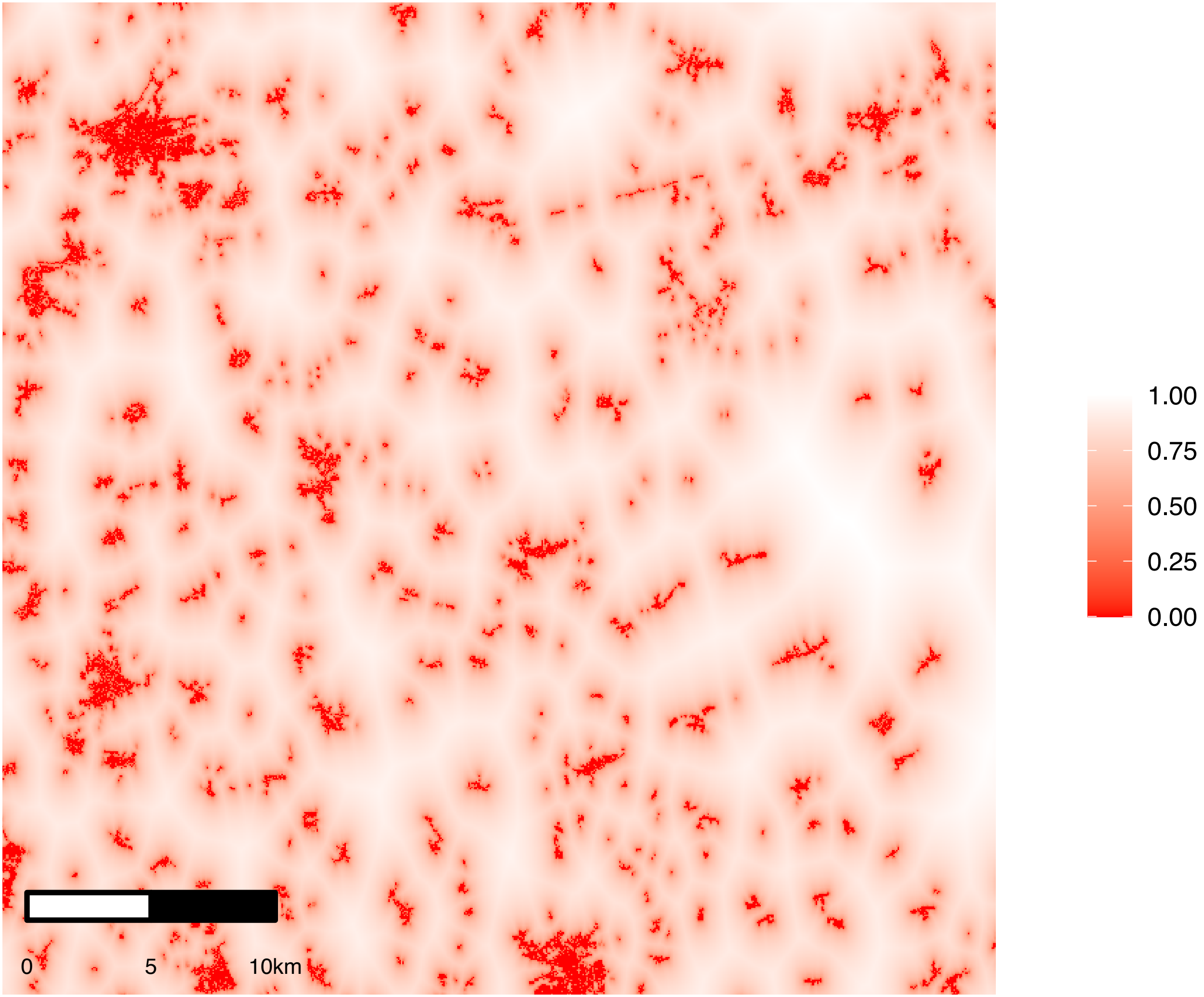

A log stretch has been applied to this layer.

Mean NDVI

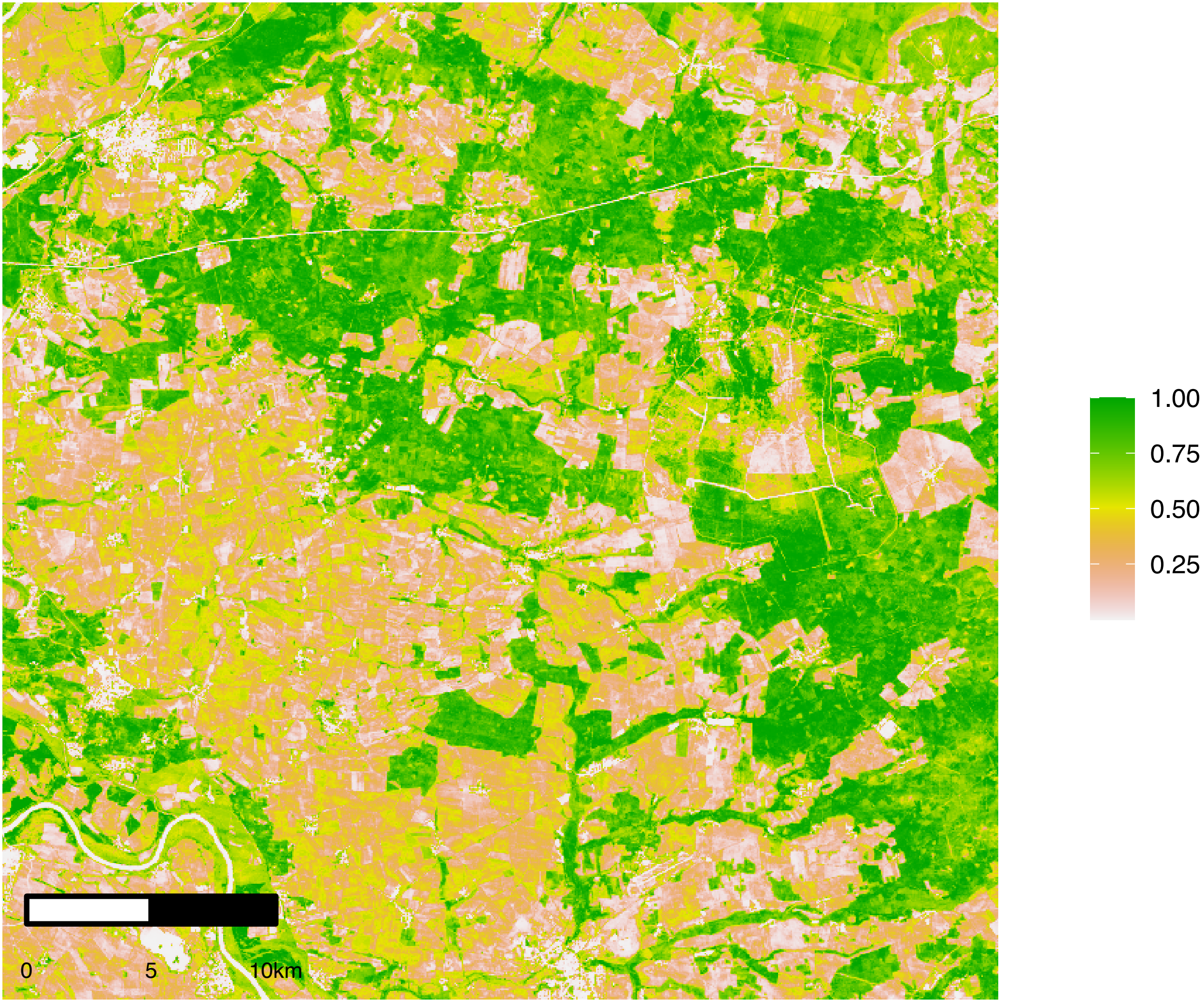

A histogram stretch has been applied to this layer.

CV of NDVI

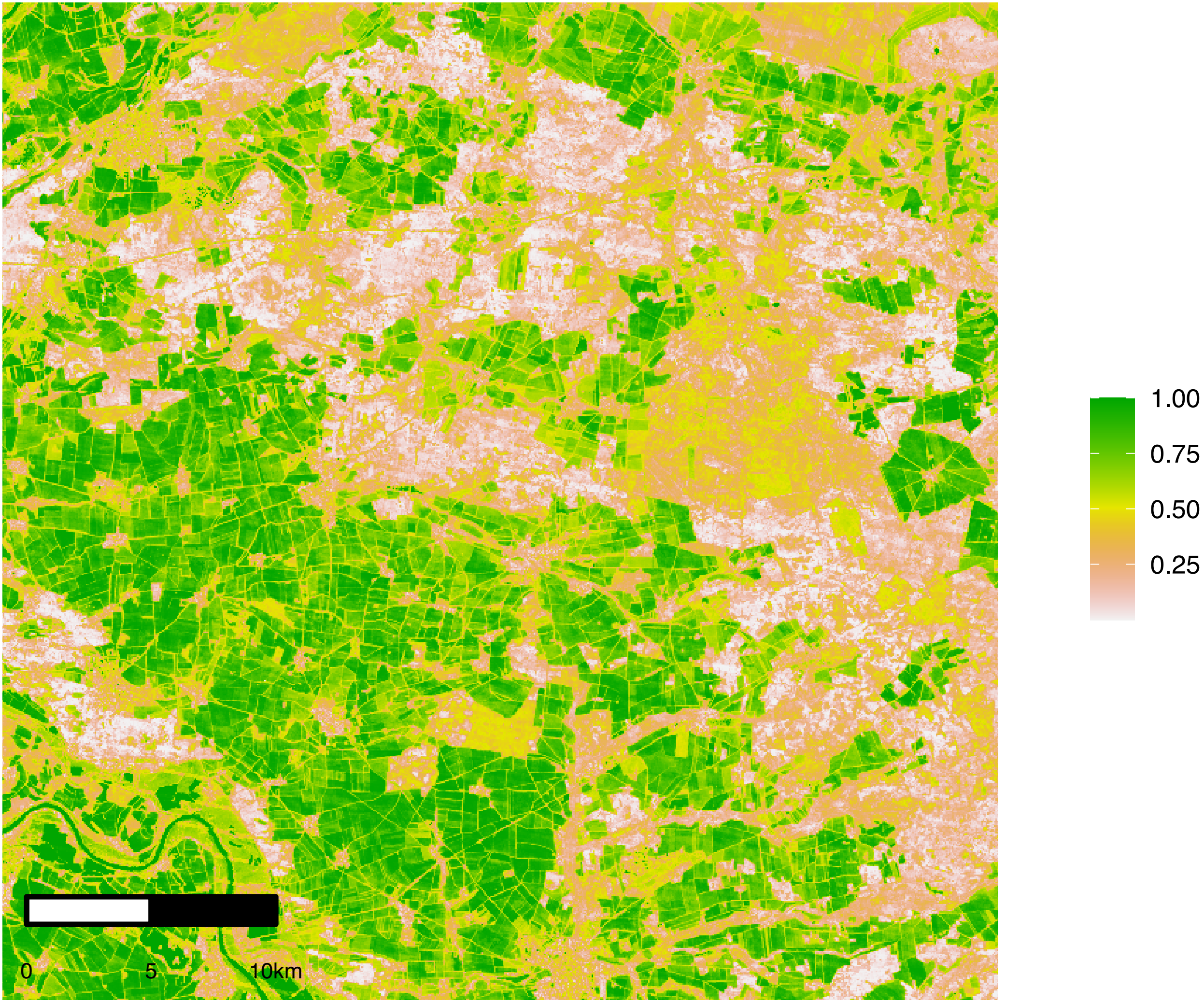

A histogram stretch has been applied to this layer.

Percent bare

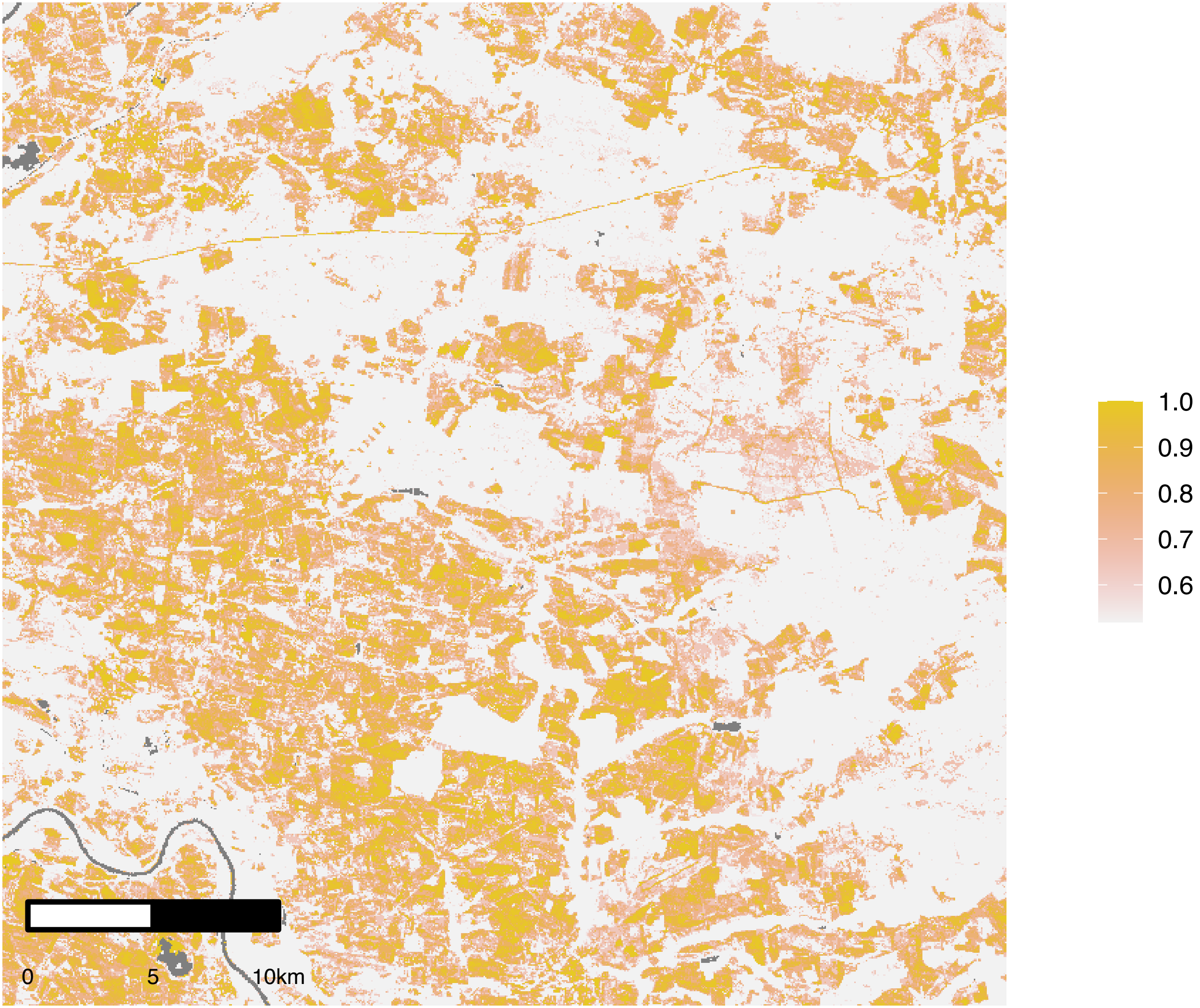

A histogram stretch has been applied to this layer.

Percent Tree

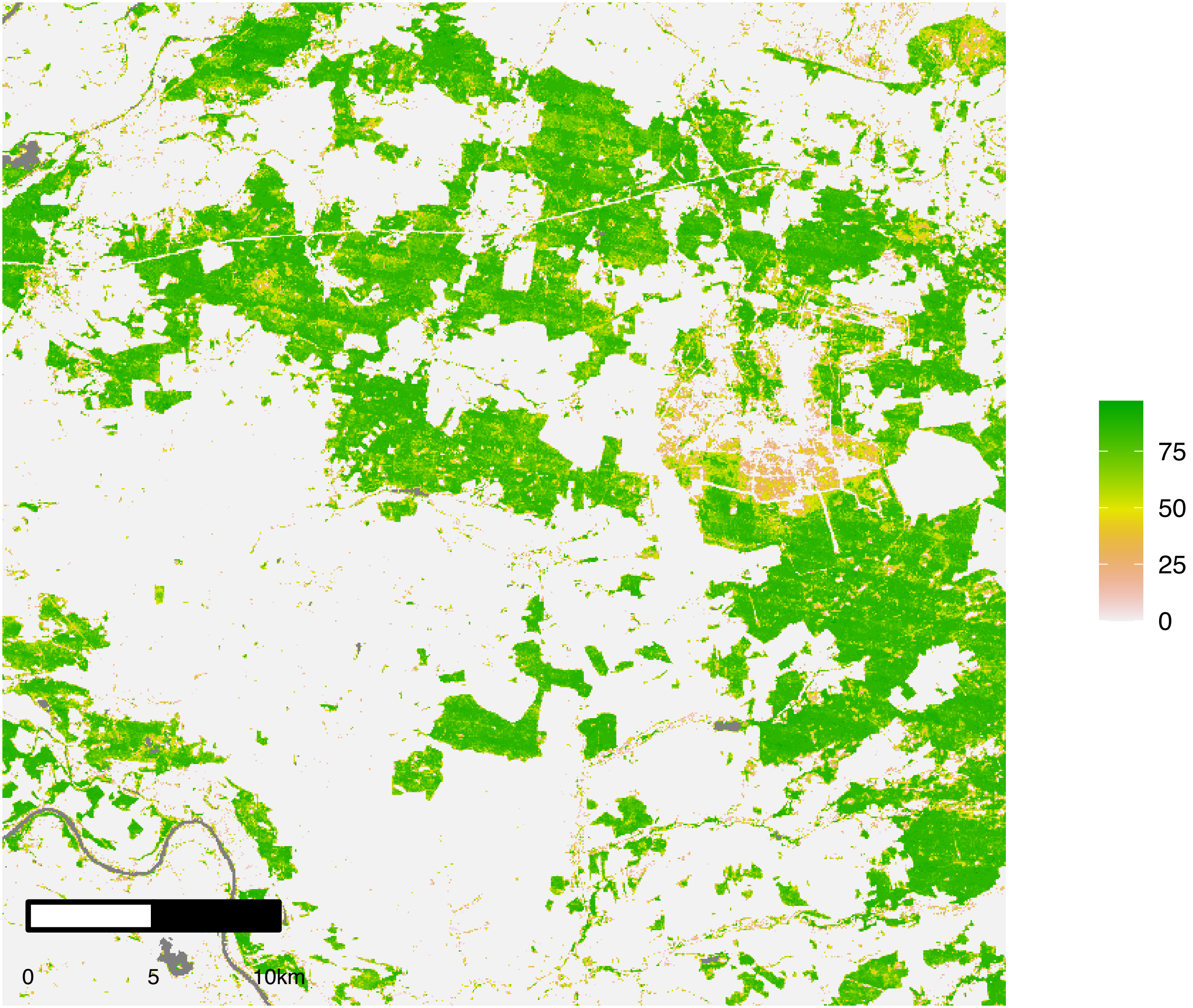

Supplementary Figure 10. Maps of the five environmental variables used in niche estimation for the Loburg population. Please see Table S1 for information about these variables. The NDVI variable is a time series (16-day), so mean and CV is presented.

References

1. Masek, J. G. *et al.* A Landsat surface reflectance dataset for North America, 1990-2000. *IEEE Geoscience and Remote Sensing Letters* **3**, 68–72 (2006).
2. Vermote, E., Justice, C., Claverie, M. & Franch, B. Preliminary analysis of the performance of the Landsat 8/OLI land surface reflectance product. *Remote Sensing of Environment* **185**, 46–56 (2016).
3. Roy, D. P. *et al.* Characterization of Landsat-7 to Landsat-8 reflective wavelength and normalized difference vegetation index continuity. *Remote Sensing of Environment* **185**, 57–70 (2016).
4. Hansen, M. C. *et al.* High-Resolution Global Maps of 21st-Century Forest Cover Change. *Science* **342**, 850–853 (2013).
5. GUF-Team. Global Urban Footprint. http://www.dlr.de/eoc/en/desktopdefault.aspx/tabid-9628/16557_read-40454/ (2018).
