# Supplementary material for: Individual environmental niches in mobile organisms
Source: Nat Commun. 2021 Jul 27;12:4572. doi: 10.1038/s41467-021-24826-x (PMC8316569; doi:10.1038/s41467-021-24826-x)
Supplement: Supplementary file 3 — Description of Additional Supplementary Files [file 41467_2021_24826_MOESM3_ESM.docx]

**Supplementary Data 1.** Mean and 95% confidence intervals for niche volume estimates. Uncertainty is based on a bootstrap approach in which we resampled, with replacement, within each individual/year one hundred times and recalculated hypervolumes. animal_name–the name of the individual; population–the population that the individual belongs to; year– the breeding year; mean–the mean volume of the bootstrap distribution for the individual/year; ci_low–the 2.5% quantile of the bootstrap distribution for the individual/year; ci_high–the 97.5% quantile of the bootstrap distribution for the individual/year.
